# RNA Editing and Retrotransposons in Neurology

**DOI:** 10.3389/fnmol.2018.00163

**Published:** 2018-05-23

**Authors:** Heinz Krestel, Jochen C. Meier

**Affiliations:** ^1^Department of Neurology, Bern University Hospital and University of Bern, Bern, Switzerland; ^2^Department for BioMedical Research, Bern University Hospital and University of Bern, Bern, Switzerland; ^3^Division Cell Physiology, Zoological Institute, Technical University Braunschweig, Braunschweig, Germany

**Keywords:** Alu element, L1 retrotransposon, A-to-I editing, neuroinflammation, genetic rearrangement, frontotemporal dementia, ALS, epilepsy

## Abstract

Compared to sites in protein-coding sequences many more targets undergoing adenosine to inosine (A-to-I) RNA editing were discovered in non-coding regions of human cerebral transcripts, particularly in genetic transposable elements called retrotransposons. We review here the interaction mechanisms of RNA editing and retrotransposons and their impact on normal function and human neurological diseases. Exemplarily, A-to-I editing of retrotransposons embedded in protein-coding mRNAs can contribute to protein abundance and function via circular RNA formation, alternative splicing, and exonization or silencing of retrotransposons. Interactions leading to disease are not very well understood. We describe human diseases with involvement of the central nervous system including inborn errors of metabolism, neurodevelopmental disorders, neuroinflammatory and neurodegenerative and paroxysmal diseases, in which retrotransposons (*Alu* and/or L1 elements) appear to be causally involved in genetic rearrangements. Sole binding of single-stranded retrotransposon transcripts by RNA editing enzymes rather than enzymatic deamination may have a homeostatic effect on retrotransposon turnover. We also review evidence in support of the emerging pathophysiological function of A-to-I editing of retrotransposons in inflammation and its implication for different neurological diseases including amyotrophic lateral sclerosis, frontotemporal dementia, Alzheimer's and Parkinson's disease, and epilepsy.

## Introduction

The complexity of humans must be due to more than the number of their genes, because a dichotomy exists between the functional complexity of some organs, such as the brain, and the number of genes in humans compared to other organisms. The molecular mechanisms of functional complexity are mannifold. It was progressively realized in recent years that epigenetic regulation, alternative splicing, post-transcriptional and -translational modifications, and in addition a somatic mosaicism of DNA, not only in protein-coding sequences but also in the large genome regions that do not code for proteins and contain amongst others regulatory functions, contribute to this complexity. One of these post-transcriptional site-specific modifications, RNA editing, enzymatically modifies specific nucleotides and changes one nucleotide into another. A set of RNA editing enzymes are adenosine deaminases that act on RNA (ADAR), which recognize adenosines present in double-stranded RNA (dsRNA) regions and catalyze the deamination of adenosine to inosine (A-to-I editing). Inosine in protein-coding transcripts is read as guanosine by the cellular translation machinery. Examples of transcripts edited by ADAR are mRNAs encoding glutamate receptors, serotonin receptors, and potassium channels in the central nervous system (CNS) (Rosenthal and Seeburg, 2012; Meier et al., 2016). Adenosines are not only deaminated in protein-coding parts, but in fact in the majority of cases in non-coding regions of sequences. The protein-coding part of the genome contains an estimated RNA editing to significant extent (>20%) of ~80 genes (Nishikura, 2016) (which might correspond to 100–150 editing sites, annotation by this review's authors), while the total number of A-to-I editing sites identified to date within the Genotype-Tissue Expression (GTEx) project are 2'802'751 with an estimated higher true positive rate (Supplementary Note 1 in Tan et al., 2017). Most editing sites reside in non-coding regions, and particularly within sequences called transposons via RNA intermediates (retrotransposons). Due to the prevalence of A-to-I editing in retrotransposons, and the strong effort so far made to elucidate the physiological and pathophysiological roles of retrotransposons regulated by RNA editing, it is timely and important to review current knowledge of these mechanisms from the point of view of neurology. Alterations in non-coding regions of the genome in the affected organs may explain low genotype-phenotype correlation (Erwin et al., 2016), and this review will focus on the human CNS by beginning with an introduction on transposable elements (TE or transposons) and the subset of retrotransposons that has as yet been described to be related to A-to-I editing and ADARs.

Transposons are frequent and make up about half of the human genome (44%; Mills et al., 2007). They can be classified into class I retrotransposons and class II DNA transposons upon their structural characteristics and mechanism of transposition (Cordaux and Batzer, 2009; Erwin et al., 2014; Kazazian, 2017). Class I retrotransposons, and in particular non-long terminal repeat (LTR) retrotransposons are regulated by RNA editing. Some remarkable characteristics should briefly be emphasized here: First, the DNA sequence of non-LTR retrotransposons contains a poly(A) tail at its 3′end with up to 60 nucleotides in length (Kazazian et al., 1988). Second, retrotransposons are transcribed by RNA polymerase II or III as non-coding transcripts with a poly(A)-tail and exported to the cytoplasm, or they are embedded in untranslated regions of RNA polymerase II generated mRNAs. As introns are spliced out prior to cytoplasmic export, retrotransposons are found in 5′UTRs and 3′UTRs of mRNAs. Lack of long terminal repeats, protein-coding capacity, and size further determine non-LTR retrotransposon classification. The first group consists of long interspersed nuclear (genome) elements (LINE). One type, LINE-1 or L1, has a full length of about 6 kb and with 500,000–700,000 copies constitutes ~15–17% of the human genome (Sela et al., 2007; Beck et al., 2010). Human-specific L1 (L1Hs) represent the only known autonomous retrotransposon family with ~80–100 active L1Hs elements per human individual (Brouha et al., 2003). Most L1 have lost their ability to retranspose probably due to accumulated mutations/fragmentation, DNA promoter methylation and heterochromatin formation, and ADAR1-dependent restriction through binding the basal L1-ribonucleoprotein (RNP) complex rather than through its editing activity (Orecchini et al., 2017a). Active L1 elements are transcribed from an internal Pol II promoter in the 5′UTR and contain two non-overlapping open reading frames ORF1 and ORF2. The latter encodes a protein with endonuclease and reverse transcriptase activities. It is generally accepted that L1 endonuclease introduces a nick at the consensus site 3′-AA/TTTT-5′ by cleaving at the bottom strand, which allows the T's at the 3′ terminus of the nick to prime reverse transcription from the poly(A) end of a L1 transcript.

The second group consists of short interspersed nuclear elements (SINE). *Alu* elements are the most prevalent non-LTR retrotransposon with >1 million copies in the human genome (Lander et al., 2001). The typical full-length *Alu* element is 300 bp, depending on the length of the 3′ oligo d(A)-rich tail. *Alu* elements are either embedded in protein-coding mRNAs or transcribed from an internal RNA polymerase III (Pol III) promoter into single-stranded RNAs (Shen et al., 1997). *Alu* elements are non-autonomous and depend on the retrotransposition molecular machinery provided by L1 elements (Dewannieux et al., 2003). Major active subfamilies responsible for novel retrotransposon insertions are *Alu*Ya5/8 and *Alu*Yb8/9. Over 90% of all A-to-I editing occurs in *Alu* elements (Athanasiadis et al., 2004; Levanon et al., 2004), and preferentially in those elements, which can form intramolecular double-stranded RNA structures. Compared to the number of editing events in *Alu* elements, A-to-I editing is rather low in L1 (<5%) (Athanasiadis et al., 2004; Chung et al., 2018).

A high density of genes, elevated GC-content, strong gene expression and inverse intron length positively correlated with high SINE density and inverse (i.e., low) LINE density (Versteeg et al., 2003). Active retrotransposons were proposed to contribute to a somatic mosaic and subsequently to phenotypic variation in health and disease of the brain (Evrony et al., 2012; Upton et al., 2015; Erwin et al., 2016). Recent work showed that novel somatic L1 insertions occurred at a rate of ~0.58–1 events per cell in both glia and neurons from human hippocampus and frontal cortex and affect at least 36% of the cells in the healthy adult brain (Erwin et al., 2016). The same work also showed that fixed L1 sequences in the genome may generate somatic copy number variants (CNV) in the brain via L1 endonuclease upregulation during neuronal differentiation. This observation corresponds well with previous work from the same laboratory, showing that 13–41% of cells from frontal cortex of healthy deceased human beings harbored at least 1 new CNV between 500 kb −1 Mb in size, with deletions being 2x more frequent than insertions (McConnell et al., 2013). The novel somatic insertion rate of *Alu* elements in the adult human brain is unknown. Neuronal retrotransposition may affect memorization (Richardson et al., 2014), which was convincingly demonstrated in case of olfactory memory formation in Drosophila (Perrat et al., 2013). In humans, a retrotransposition and DNA editing-dependent process of memory encoding and storage was previously suggested but not proven yet (Mehler and Mattick, 2007; Mehler, 2008).

## Data base research

A systematic review of primary and secondary literature was performed here to study and discuss A-to-I editing and retrotransposons, their functions and interactions in the human CNS in physiological conditions and disease. PubMed was searched using the keywords “RNA editing” or “A-to-I editing” together with “review”, “human brain”, “micro RNA”, “piwi RNA”, “(endo-)siRNA”, “gene expression”, and “3′-UTR.” “5′-UTR”, “intron” that cover non-coding pre-mRNA structures. Further search terms were “ADAR”, “ADAR, brain”, “DNA editing, ADAR”, and “ADAR, disease”. Furthermore, PubMed was searched using keywords “retrotransposon” or “LINE” or “SINE” or “Alu” in combination with “incidence”, “review”, “human brain”, “health”, “disease”, “RNA editing”, “A-to-I editing”, “ADAR”, “ADAR1”, “ADAR2”, “APOBEC”, “(endo-)siRNA”, “brain tumor”, extracerebral cancers (such as lung and colorectal cancer), “epilepsy”, and “TDP-43”, Literature search for *known mechanistic interactions* of RNA editing with retrotransposons was performed using the keywords “RNA editing”, “A-to-I editing”, “retrotransposon”, “Alu”, “L1” together with “translational efficacy”, “gene expression”, “gene expression, brain”, “exonization”, “exon evolution”, “backsplicing”, “silencing”, and “circular RNA”. The investigation of potential immunogenicity of deficiently edited inverted-repeat *Alu* elements in 3′-UTR of mRNAs was done in the context of above-mentioned keywords together with “3′-UTR”, “inflammation”, “immunogenicity”, “immune system”, “inflammasome”. *Physiological* functions of retrotransposons and RNA editing in the CNS were investigated by searching the PubMed database using keywords “brain development”, “cognition”, “learning and memory”, “memory”, “neurogenesis”, and “intelligence”. *Pathophysiological* functions of RNA editing and transposable elements were investigated for all the diseases listed in Table 1 and Supplementary Table 1, together with the keywords “Alu”, “RNA editing”, and “ADAR”. The mechanisms of copy number variants (CNV) were also addressed.

**Table 1:** Neurological diseases with primary genetic and/or neurodegenerative origin.

**Disease**	**Transposable elements (human data)**	**RNA editing (human data)**	**Disease genes**
Friedreich ataxia	Expanded GAA triplet repeat in central *Alu* linker located in *FXN* intron 1 with allelic suppression	Not known	*FXN* (9q)
Ataxia teleangiectasia	*Alu*-mediated exonization in *ATM* intron 20 (Pagani et al., 2002); increased genomic L1 copy number in hippocampi from ataxia teleangiectasia patients (Coufal et al., 2011)	Not known	*ATM* (11q)
ALS type 12	5 patients with different types of *Alu*-mediated *OPTN* exon deletions (Maruyama et al., 2010; Iida et al., 2012)	Deficient GRIA2 Q/R editing in spinal motor neurons (Kawahara et al., 2004); Deficient GRIA2 Q/R editing suggested to contribute to the generation of TDP-43 positive cytoplasmic inclusion bodies (Yamashita et al., 2017)	*OPTN* (10p) See OMIM for further ALS candidate genes
Frontotemporal dementia (FTD)	Unproven *Alu*-mediated deletion of *OPTN* exons 13–15 (Pottier et al., 2015)	Not known	*OPTN* (10p), *MAPT, PSEN1, GRN, TARDBP*, and others
ALS-FTD		See ALS and FTD	*C9ORF72* (9p)
Hereditary spastic paraplegia	*Alu*-mediated rearrangement of *SPAST* in ~60 patients (Boone et al., 2011, 2014; Jahic et al., 2016); 1 patient with *Alu*-mediated deletion of SPG7 exons 11–13 (López et al., 2015); 3 patients with *Alu* microhomology-mediated exon deletion in *SPG11* (Conceição Pereira et al., 2012)	Not known	*SPAST* (2p) *SPG7* (16q), *SPG11* (15q)
Parkinson disease	1 patient with *Alu*-mediated *PARK2* exon 10 deletion (Morais et al., 2016); 1 patient with *Alu*-mediated *DJ1* exon 1-5 deletion (Bonifati et al., 2003)	Not known	*PARK2* (6q), *DJ1* (1p); for other genes see OMIM
Alzheimer disease	1 patient with *Alu*-mediated *PSEN1* exon 9-10 deletion (Le Guennec et al., 2017)	A-to-I editing reduced at multiple sites in prefrontal cortex and hippocampus (see main text; Akbarian et al., 1995; Gaisler-Salomon et al., 2014; Khermesh et al., 2016)	*APP, PSEN1* (14q), *PSEN2*
Epilepsy	5 patients with *Alu*-mediated rearrangement of portions of *ALDH7A1* (Mefford et al., 2015); 3 patients with *Alu*-mediated *CDKL5* deletions of various sizes (Erez et al., 2009)	Reduced and elevated A-to-I editing at various sites (e.g. Krestel et al., 2013)	*ALDH7A1* (5q), *CDKL5* (Xp) See OMIM for further epilepsy candiate genes
Aicardi-Goutières syndrome (AGS)	ADAR1 primarily edits *Alus* in RNA Pol II transcribed mRNAs, ADAR1 ko neuronal progenitor cells exihibit spontaneous IFN production, inhibition of mRNA translation, and apoptosis (Chung et al., 2018)	Reduced A-to-I editing of miR376-a2 by *ADAR1* mutation frequently found in Aicardi-Goutières syndrome (Rice et al., 2012)	*ADAR1* (1q), *SAMHD1, TREX1, IFIH1*

*Diseases, for which human data about genetic rearrangements in association with transposable elements have been identified. For these diseases, also human data about RNA editing were investigated and are presented. RNA editing has an important function in Aicardi-Goutrières syndrome and thus it is presented here, although transposable elements have as yet not been causally related with its development. The very right column of the table contains major candidate genes (with chromosomal location given in brackets) that have been implicated in the respective diseases*.

## Retrotransposons and RNA editing in normal brain function

Genomic deletions with transposable element (TE) involvement have been identified, which presumedly take place in the genome of neural progenitor cells (Erwin et al., 2016). Actually, L1 retrotransposition has already been demonstrated in neural progenitor cells (Coufal et al., 2009), suggesting that it can occur during adult neurogenesis in the hippocampus and subventricular zone. It remains however to be clarified, whether retrotransposition of L1 and *Alu* elements can occur in postmitotic neurons and glia and contributes in this way to phenotypic diversity.

A-to-I editing levels at protein-coding sites and repetitive (typically non-coding) sites were overall comparable in various human tissues (including the brain) of >500 individuals from the GTEx project. Regarding editing in coding sites, cells that make up arteries had the highest rates, while muscle cells showed the lowest editing rates. Editing in repetitive sites was highest in arteries and cerebellum, while muscle again showed the lowest editing rates (Tan et al., 2017). In the following subsections, interactions between retrotransposons and A-to-I editing at the DNA, pre-mRNA, and mRNA levels will be reviewed and additionally revealed whether such mechanisms have already been demonstrated for human brain cells.

### At the DNA level

In the Drosophila nervous system, drosophila ADAR (dADAR) can bind to a chromosomal locus containing a gene that carries a transposon with tandem repeats, called Hoppel element, in one of its introns (Savva et al., 2013). A long dsRNA is generated from this transposon that can form perfectly matched double-stranded RNA, if not edited by dADAR. Deficient or missing A-to-I editing of the Hoppel transposon can initiate silencing of other genes and transposons in trans. A similar mechanism of gene regulation by editing of single-stranded TE transcripts has not been reported in mammalian cells.

### At the pre-mRNA level

66% of *Alu* elements and 58% of L1 are located in introns of the human transcriptome (Sela et al., 2007). Over 25% of protein-coding human mRNAs contained retrotransposons in their 3′UTR, ~6% carried *Alu* elements, and 5% L1 (Faulkner et al., 2009).

**Figure 1:**
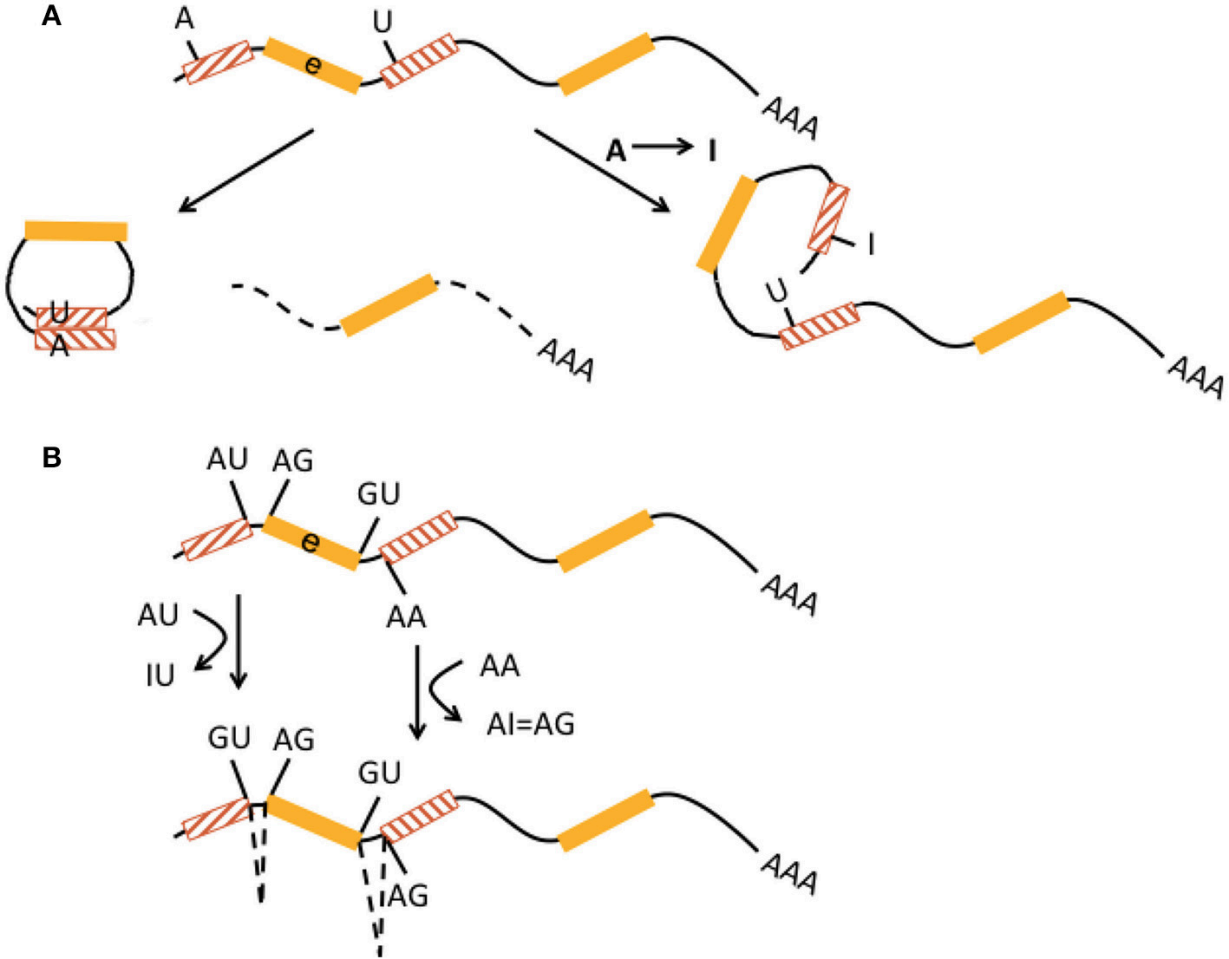
Interaction of RNA editing and retrotransposons at the pre-mRNA level. **(A)** Non-LTR retrotransposons in inverted orientation in introns (hatched boxes with red lines pointing in opposite directions) flanking an exon (e, yellow boxes) may base-pair into double-stranded RNA and form circular RNA (left) by a mechanism called backsplicing. The residual pre-mRNA is subsequently degraded (dotted line). Deamination of an adenosine to an inosine (A → I) by ADAR within a non-LTR retrotransposon may prevent perfect base pairing (right) and hence formation of double-stranded cirular RNA and backsplicing. This pre-mRNA may be regularly spliced and exported. Both mechanisms may contribute to regulation of translational efficiency. **(B)** A-to-I editing can activate cryptic splice sites in non-LTR retrotransposons (hatched boxes) by deamination and creation of new splice donor (AU edited to IU = GU) and acceptor (AA edited to AI = AG) sites, resulting in alternative splicing and exonization of non-LTR retrotransposons. This may lead to new gene functions.

Non-LTR retrotransposons were proposed to contribute to the regulation of transcriptional efficiency/protein abundance by their ability to form double-stranded (ds) RNA structures. Through a mechanism called backsplicing, circular RNAs (circRNA) are generated from pre-mRNAs that contain complementary *Alu* elements in inverted orientation in introns flanking exons (Figure 1A). A-to-I RNA editing may prevent perfect dsRNA formation of inverted *Alu* elements and thus suppress backsplicing. Up to 15% of actively transcribed genes generate circRNAs and some genes seem to produce 10 times more circular than linear RNA. CircRNAs cannot be exported into the cytoplasm and thus reduce respective gene expression (Ivanov et al., 2015; Wilusz, 2015). CircRNAs are highly abundant in the human brain. A catalog of neuronal circRNAs was generated using human data from the ENCODE project and involving neurons that were differentiated from neuroblastoma cell lines. CircRNAs were indeed shown to be upregulated during neuronal differentiation and after ADAR1 siRNA knockdown in neuroblastoma cells (Rybak-Wolf et al., 2015).

A-to-I editing of intronic *Alu* elements may also lead to the generation of new splice donor (AU edited to IU = GU) and acceptor (AA edited to AI = AG) sites and trigger alternative splicing and integration of *Alu* elements into exons that can result in novel gene functions (keyword “exonization,” Figure 1B) (Lev-Maor et al., 2007; Möller-Krull et al., 2008; Nishikura, 2016). A well-known example is auto-editing of the human *ADARB1* (*ADAR2*) gene transcript by its protein, in which intronic AA dinucleotides turn into a functional AG 3′ splice site and alternative splicing with *Alu* exonization (into exon 8) creates a 2-fold difference of this gene product's editing activity. ADARB1 is strongly expressed in the brain (Gerber et al., 1997; Sela et al., 2007). Another example is *Alu* exonization via A-to-I editing in pre-mRNA of the human nuclear prelamin A recognition factor (NARF) that is ubiquitously expressed including brain. Two *Alu* elements in *NARF* intron 7 form a dsRNA structure and undergo RNA editing, which generates a new slice acceptor site, abolishes a stop codon, and leads to (pseudo)exon 8 (Lev-Maor et al., 2007). However, exonization by A-to-I editing appears to be a rather rare event (Athanasiadis et al., 2004). It is estimated that only 4% of all transposon-derived exons contribute to new functionality of the proteome (Sela et al., 2007). Exonisation of *Alu* elements can also occur in 5′UTRs and generate new upstream open reading frames (Shen et al., 2011). Alu exonization into the 3′UTR was described to occur by RNA editing-mediated generation of a splice donor site in transcripts from the *GPR81* gene, a G protein-coupled receptor with some expression in the brain (Athanasiadis et al., 2004).

mRNAs carrying retrotransposons in their 3′UTRs appear to be less expressed than mRNAs without retrotransposons. In this regard, several mechanisms were discussed involving interference with non-coding transcripts from 3′UTRs or with other cis-regulatory elements, introduction of miRNA binding sites, truncation of 3′UTRs by premature polyadenylation signals in retrotransposons, and degradation in *trans* by other retrotransposons (Faulkner et al., 2009). Experiments in HeLa and HEK293 cells proposed that human mRNAs with inverted-repeat *Alu* elements in their 3′UTR were as likely exported to the cytoplasm as mRNAs lacking these structures, but they were in most cases translated less efficiently. Effects on translation were independent of the presence of inosine – i.e., they were sequence- (and thus RNA editing) independent - but rather dependent on the distance of the dsRNA structure from the stop codon (Capshew et al., 2012).

**Figure 2:**
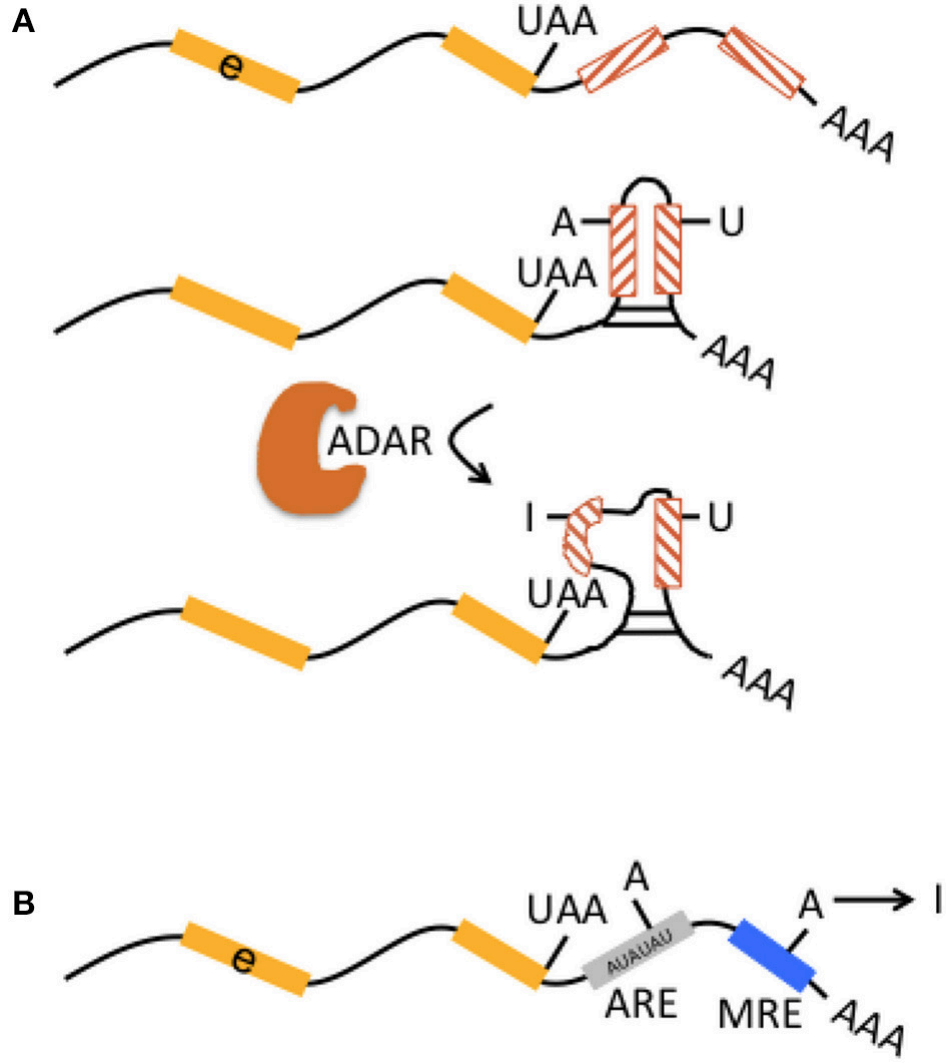
Interaction of RNA editing and retrotransposons at the pre-mRNA level. **(A)** Multiple non-LTR retrotransposons may be present in 3′UTR [indicated by preceding exon (e, yellow boxes) with stop codon “UAA”] in inverted orientation (lines in hatched boxes point in opposite directions). They may form perfectly matched double-stranded RNA structures (middle graph). Inverted-retrotransposon double-stranded RNA structures may also undergo A-to-I editing by ADAR, resulting in loop formation within double-stranded RNA (lower graph). The formation of partially matched double-stranded RNA structures in the 3′UTR is implicated in escape from cytosolic immune response. **(B)** AU-rich elements (ARE, gray box) and microRNA response elements (MRE, blue box) in 3′UTRs represent binding sites for proteins and microRNAs that can affect mRNA stability and translational efficiency. A-to-I editing may modulate adenosines within ARE and MRE and thus indirectly influence those mechanisms.

Introns and 3′UTRs may harbor multiple *Alu* elements that are located in inverted orientation. Inverted *Alu* elements can form intramolecular dsRNA structures and undergo A-to-I editing (Figure 2A; Capshew et al., 2012). The function of edited *Alu* elements in Pol II-transcribed mRNAs has now been impressively demonstrated in human cells including HEK293 cells and neuronal progenitor cells (Chung et al., 2018). In more detail, *Alu* elements are the primary editing target of ADAR1 (91%) in HEK293 cells. ADAR1-edited *Alu* elements were mainly found in introns (48%) and 3′ UTRs (37%). Very few (<0.1%) of ADAR1 edits were found in open reading frames and corresponding genes (i.e., their pre-mRNAs) edited by ADAR1 showed no significant enrichment of any gene ontology terms. About 5% of other TEs including L1 are editing targets of ADAR1. Actually, ADAR1 primarily edits *Alu* elements in RNA Pol II transcribed mRNAs, not Pol III transcribed *Alu* transcripts. ADAR1 editing does not affect mRNA abundance. Binding of ADAR1 to dsRNA structures within mRNAs as well as A-to-I editing prevents inhibition of both efficient translation of other mRNAs and cell proliferation, which are otherwise triggered during an inflammatory interferon-γ (IFN) response. Differentiation of ADAR1-deficient human embryonic stem cells to neuronal progenitor cells leads to spontaneous IFN production, activation of protein kinase R, and apoptotic cell death. Melanoma differentiation-associated gene 5 (MDA5 or interferon-induced helicase C domain containing protein 1, IFIH1), a soluble RNA receptor is the major dsRNA sensor for induction of an IFN-mediated inflammatory response and ADAR1 is a key player in preventing spontaneous MDA5 activation. MDA5 is particularly activated by the dsRNA structures formed by inverted-repeat *Alus*, which are largely present in 3′ UTR of retrotransposition-incompetent Pol II transcripts and not by the unpaired *Alu* elements from retrotranspostion-competent Pol III transcripts. Thus, deficient A-to-I editing of inverted-repeat *Alus* may “breach” the immune tolerance of MDA5 (Ahmad et al., 2018).

Finally, A-to-I editing may also affect AU-rich elements (ARE) and micro RNA response elements (MRE) in the 3′UTR (in mice; Liddicoat et al., 2015), thereby contributing to regulation of mRNA stability and translational efficiency (Figure 2B).

### At the mRNA level

**Figure 3:**
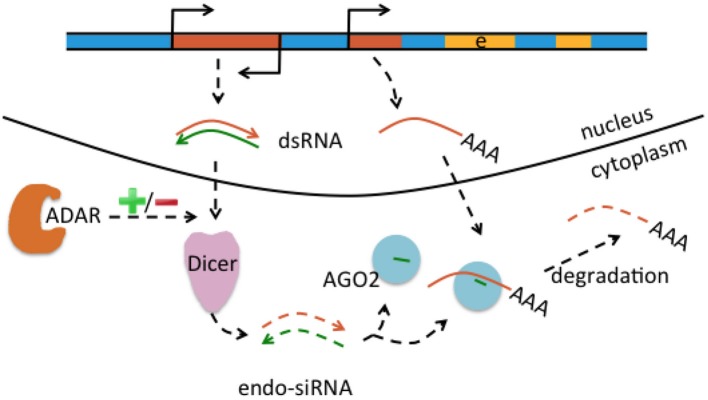
Interaction of RNA editing and retrotransposons at the mRNA level. Retrotransposons (brown boxes) may be transcribed in sense and antisense direction (indicated by promoter symbols pointing in opposite directions), form double-stranded RNA (dsRNA) precursors (brown and green arrows) that are processed by Dicer into endo short interference RNAs (endo-siRNA). Endo-siRNAs (here indicated as short green lines) are loaded onto AGO2 proteins, may detect further single-stranded retrotransposons transcribed from other locations (middle brown brown box) and target them for degradation. Thus, the siRNA pathway contributes to retrotransposon silencing. Levels of endo-siRNA can be affected by ADAR and/or A-to-I editing, with an increase or decrease (indicated by “+/–“) of small RNA upon loss of ADAR.

Genomic *Alu* and L1 elements serve as templates for small non-coding RNAs such as piRNAs and endo-siRNAs that themselves do not affect the expression of protein-coding genes but control and quiesce autonomous L1 and *Alu* RNAs (Figure 3; Saito and Siomi, 2010). ADARs can have an effect on the level of small non-coding RNAs. This is supported by ADAR knockouts in *Caenorhabditis elegans* that notably had little influence on the nucleotide sequence of mature miRNA, endo-siRNAs, and piRNAs, but on the contrary changed expression levels of the majority of small RNAs up to 40% when compared to wildtype (Warf et al., 2012). While previous studies suggested an increase of endo-siRNA levels in the absence of ADAR function (e.g., Wu et al., 2011), this study found predominantly reduced small RNA levels in ADAR mutants (Warf et al., 2012). The authors furthermore discussed an A-to-I editing impact on dsRNA structures in the context of small RNA biogenesis or an RNA editing-independent sequestration of dsRNA by ADAR (Figure 3). Thus, normal ADAR protein abundance and A-to-I editing may contribute to the suppression of retrotransposons; a mechanism that remains to be shown in human differentiated neurons and glia.

## Retrotransposons and RNA editing in neurological disease

L1 and *Alu* elements have also been linked to disease. Several comprehensive reviews on retrotransposons and specific disease conditions have been published (Ostertag and Kazazian, 2001; Chen et al., 2005; Belancio et al., 2008; Hancks and Kazazian, 2012; Kaer and Speek, 2013; Elbarbary et al., 2016; Larsen et al., 2018). We are trying to provide here a comprehensive synergy of mechanisms involved in the diseases with CNS involvement associated with TEs (as listed in Table 1 and Supplementary Table 1). The Supplementary Text provides further detailed information.

### Spinocerebellar ataxias

Spinocerebellar ataxias are a group of neurodegenerative genetic disorders characterized by slowly progressive incoordination of gait and often associated with poor coordination of hands, speech, and eye movements. Spinocerebellar ataxias can be subdivided frequently according to other non-cerebellar features including parkinsonism, chorea, pyramidal signs, cognitive impairment, peripheral neuropathy, and seizures. *Friedreich ataxia* is the best known and most commonly inherited form of spinocerebellar ataxia. It can be caused by mutations or, in 98% of cases, by GAA trinucleotide-repeat expansions located at the center of an *Alu*Sq element in intron 1 of the frataxin (*FXN*) gene. Friedreich ataxia is the only known disease caused by abnormal expansion of a GAA trinucleotide-repeat sequence. It was suggested that GAA repeats arose by mutation or A-to-G conversion from poly(A) tracts of *Alu* elements. Many longer GAA repeats in the human genome can be found in the 3′ poly(A) tracts of *Alu* elements, but it was suggested that A-to-G conversion that led to poly-GAA repeats in Friedreich ataxia arose in the central linker region of *Alu* elements (Clark et al., 2004). Beyond GAA repeats, *Alu* elements were in general described to be a source for microsatellites (Arcot et al., 1995). Expansion of trinucleotide repeats was proposed to have arisen in Friedreich ataxia rather by in-tandem duplication up to a certain repeat length (Clark et al., 2004; Monticelli et al., 2004). From a certain repeat length onwards, genetic instability was proposed to contribute to additional repeat expansion, which is known in Neurology as anticipation. GAA repeat expansions affect pre-mRNA processing by inducing the accumulation of upstream splicing intermediates (Baralle et al., 2008). No interaction of RNA editing with these genetic rearrangements in Friedreich ataxia has been reported (Table 1).

### Ataxia teleangiectasia

Ataxia teleangiectasia is a variant spinocerebellar ataxia associated with immunodeficiency, blood vessel dilatation, hypogonadism, premature aging, genomic instability, radiation sensitivity, and cancer predisoposition. It can be caused by over 400 mutations (see Online Mendelian Inheritance in Man, OMIM; www.omim.org) in the *ATM* gene. ATM protein is a member of the phosphatidylinositol 3-kinase family that respond to DNA damage by phosphorylating key substrates involved in DNA repair and/or cell cycle control. Neurological manifestiations typically occur with homozygous or compound heterozygous mutations, while the cancer risk is already increased with heterozygous mutations (OMIM). 48% of the mutations in *ATM* were reported to be splicing-affecting mutations (Pastor et al., 2009). An individual with ataxia teleangiectasia was compound heterozygous for a c.2250 G-to-A splicing mutation and a 4 nt deletion in *ATM* intron 20, which were both considered to be causal (Pagani et al., 2002). The 4-base pair (bp) deletion disrupted an intronic splicing processing element, leading to activation of both an adjacent cryptic splice site and a cryptic exon. The splicing intermediate was only further processed, i.e., the cryptic exon was only included in the mRNA, if particular splicing factors bound in trans to a downstream intronic splicing enhancer that was located within an antisene *Alu* element (Pastor et al., 2009; Pastor and Pagani, 2011). Not only *Alu* elements but also L1 were reported to be involved in Ataxia teleangiectasia. A significantly increased L1 copy number [normalized to non-mobile repetitive DNA sequences (SATA, HERVH, 5sRNA gene)] was found with Taqman-based quantitative real-time PCR in postmortem hippocampal samples from 7 Ataxia teleangiectasia patients compared to hippocampi from deceased controls (Coufal et al., 2011). Yet it remains unclear how the increased L1 copy number contributed to the cause or progression of ataxia teleangiectasia in these patients.

### Amyotrophic lateral sclerosis

Amyotrophic lateral sclerosis (ALS) is a neurodegenerative, genetic disease of the upper and lower motor neuron in the cerebral cortex and spinal cord. Over 20 ALS forms have been described in association with mutations in several genes, which can as yet only explain a minority of all the ALS cases (Zou et al., 2016). The majority of ALS cases occur sporadically and up to 10% are familial. ALS type 12 with autosomal dominant or recessive inheritance can be caused by homozygous or heterozygous mutations in the optineurin (*OPTN*) gene (Maruyama et al., 2010). Two Japanese siblings with ALS 12 were reported with homozygous *OPTN* exon 5 deletion. Sequencing of the breakpoint junction showed the 5′ part to be of *Alu*Jb from intron 4 and the 3′ part to be of *Alu*Sx from intron 5 with an overlapping 12-bp microhomology. *Alu*-mediated recombination was proposed as deletion mechanism (Maruyama et al., 2010). The identical homozygous deletion with *Alu*-involvement was found in another ALS patient from a Japanese cohort. Further 3 patients of this cohort carried heterozygous deletions of exons 3–5 that occurred between *Alu* elements in introns 2 and 5. A fifth patient carried a heterozygous deletion between an *Alu* element 86 kb upstream to exon 1 and an *Alu* element in *OPTN* intron 4. All types of deletions were predicted to cause null alleles and were therefore considered to be causal (Iida et al., 2012; Table 1). One histological hallmark of ALS are neuronal protein aggregates in the form of cytoplasmatic inclusions that contain several ubiquitinated proteins including TDP-43. TDP-43 is a predominantly nuclear RNA/DNA-binding protein with functions in RNA processing and metabolism, including RNA transcription, splicing, transport, and stability. Hypoediting of the glutamine/arginine (Q/R) site of -amino-3-hydroxy-5-methyl-4-isoxazole propionic acid (AMPA) receptor subunit GRIA2 with increased intracellular Ca^2+^ influx was proposed to activate the Ca^2+^-dependent cysteine protease calpain, resulting in cleavage of TDP-43 and thereby contributing to cytoplasmic TDP-43-positive inclusions (Yamashita et al., 2017). It is an ongoing discussion whether cytoplasmic inclusions are beneficial or toxic to neurons. Beyond protein aggregates, neuronal excitotoxicity has been implicated in ALS. One mechanism of neuronal hyperexcitability was shown to be increased Ca^2+^ influx through deficiently edited GRIA2-containing AMPA receptors (by ADAR2) that contributed to the death of spinal motor neurons (Kawahara et al., 2004; Table 1).

### Frontotemporal dementia

**Figure 4:**
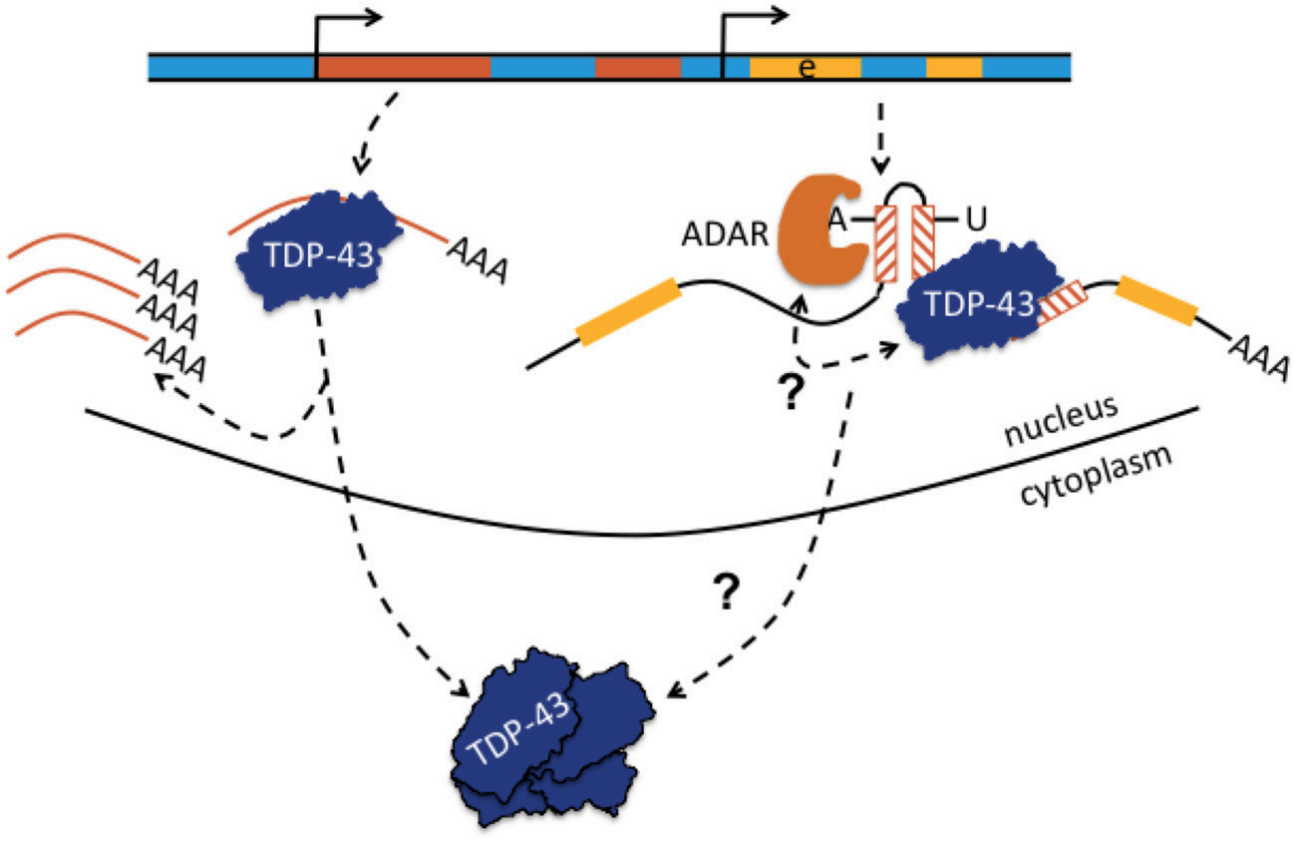
Transactive response DNA-binding protein (TDP-43 or TARDBP) may bind to single-stranded retrotransposons or to retrotransposons embedded in pre-mRNA and may regulate TE abundance or affect A-to-I editing. Normally, TDP-43 is proposed to act as a scavenger of TE-derived transcripts and regulates TE abundance. When TDP-43 function becomes comprised, as in certain neurodegenerative diseases with depletion of TDP-43 from the nucleus and aggreation in the neuronal cytoplasm, TEs become overexpressed (shown left-hand; Krug et al., 2017). Depicted right-hand, TDP-43 can also bind to retrotransposons contained in pre-mRNA and it was proposed that TDP-43 limits the extent of ADAR-mediated A-to-I editing in intronic inverted repeat dsRNA structures. A-to-I editing increases if nuclear TDP-43 is depleted. The bidirectional arrow questions whether the interaction is reciprocal and whether altered A-to-I editing may also lead to compromised binding of TDP-43 to pre-mRNA with loss of nuclear TDP-43 predominance, translocation to the cytoplasm, and aggregation. Yellow parts represent exons (e).

Frontotemporal dementia (FTD) is one of the most frequent neurodegenerative dementias (besides Alzheimer disease) and can clinically be subdivided into behavioral variant FTD (e.g., change of personality, hyperorality, emotional lability), primary progressive aphasia (non-fluent speech, paraphasia, errors in syntax), and semantic variant dementia (loss of knowledge about function of objects, agnosia). Additional clinical symptoms can include a dysexecutive syndrome, urine incontinence, sometimes an extrapyramidal syndrome or pyramidal syndrome (signs of first and second motor neuron, i.e., symptoms of ALS). Most patients with FTD have intracellular inclusion bodies. Clinical cases are further classified according to neuro/histopathological criteria in FTD with inclusion bodies lacking histological characteristics; FTD with Tau-positive inclusion bodies, TDP-43 positive inclusion bodies, fused-in-sarcoma (FUS) or SOD-positive inclusion bodies, and others. The majority of cases carries TDP-43- and to a lesser extent Tau-positive inclusion bodies. Prevalent genes implicated in FTD are *MAPT, PSEN1, GRN, TARDBP*. Mutations in *OPTN* are rarely associated with FTD. Recently, a FTD patient with a primary progressive aphasia phenotype was identified, who carried a compound heterozygous genetic rearrangement consisting of a non-sense mutation in the *TBK1* gene and a deletion spanning *OPTN* exons 13–15. The breakpoint junction showed a microhomology domain of 25 bp. Recombination occurred between sequences contained in introns 12 and 15 (Pottier et al., 2015). Although not explicitly stated, this type of deletion is reminiscent of non-homologous end-joining or microhomology-mediated end-joining possibly with *Alu* involvement, particularly as *OPTN* was described to contain 38 *Alu* repeats (Iida et al., 2012). It would be worthwile to re-analyze the sequence data of Pottier et al. regarding the question whether the breakpoints occurred in putative *Alu* elements. The genetic rearrangement was proposed to be causal, because strongly reduced mRNA and protein expression were found in the brain of this patient (Pottier et al., 2015). The observation of *OPTN* being a candidate gene for both ALS and FTD underlines previous observations that ALS and FTD are part of a clinical, histological, and genetic disease spectrum (Neumann et al., 2006). There is also the entity ALS- FTD that combines symptoms of both diseases and is frequently associated with mutations in the *C9ORF72* gene. Intronic hexa-nucleotide repeat expansions in *C9ORF72* cause poly glycine-arginine aggregations that can be found in cytoplasmic inclusions, often together with TDP-43. ALS and FTD are subsumed into the group of TDP-43 spectrum disorders. Increased transcript levels of *Alu*YK12 elements have been found in frontal cortex of deceased patients with ALS-FTD compared to cortex from healthy deceased controls, suggesting increased retrotransposon pathology in ALS-FTD cases (Prudencio et al., 2017). Retrotransposon and transposon transcripts may also interact with TDP-43. Mining of a series of deep sequencing datasets of protein-RNA interactions (CLIP-seq) from human healthy and FTLD (frontotemporal lobar degeneration, the neuropathological term of FTD) brain tissues uncovered extensive binding of TDP-43 to TE transcripts (non-LTR, LTR, DNA elements; Li et al., 2012). The association between TDP-43 and TE-derived RNA targets was significantly reduced in patients with FTLD compared to healthy subjects. Yet the reason remained unclear particularly whether the reduced binding of TE transcripts in FTLD tissue was due to immunoprecipitation of smaller amounts of TDP-43 protein bound to TE RNA because residual TDP-43 was lumped together in cytoplasmic inclusions. Simultaneously, RNA-seq experiments in mice with TDP-43 pathology (one mouse strain with transgenic overexpression of human TDP-43; second mouse strain with antisense oligonucleotide-mediated depletion of TDP-43 in striatum that is a part of basal ganglia) both revealed significant elevation of TE-derived transcripts with a striking concordance to those TE RNAs that were identified as RNA targets of TDP-43 in CLIP-seq experiments of mice (Li et al., 2012). The hypothesis was brought forward that TDP-43 acts as a scavenger for TE-derived RNAs and elevated expression of TE-derived transcripts would occur, if TDP-43 was dysfunctional or misrepresented in the nucleus, (Li et al., 2012; Erwin et al., 2014; Figure 4). It remained to be clarified how elevated expression of TE RNA resulted in neurodegenerative disease and whether an increased number of somatic retrotransposition events occurred: in this regard, genomic instability, DNA damage, and toxic effects were discussed (Li et al., 2012). In subsequent work by the same group, it was shown that TDP-43 interfered with siRNA-mediated silencing of TE transcripts and that elevated expression of the LTR-retrotransposon gypsy (most active TE in Drosophila) was associated with enhanced TUNEL staining, taken together as evidence for increased apoptosis. In addition, experiments demonstrated that these mechanisms contributed to the ALS-phenotype in a Drosophila (Krug et al., 2017). It remains to be shown in humans, if, and if so how, increased levels of TE transcripts may contribute to ALS and FTD. RNA editing has not yet been associated with FTD or ALS-FTD, although ALS was associated with impaired editing, and FTD as well as ALS share a common pathophysiological spectrum (Table 1).

### Hereditary spastic paraplegias

Hereditary spastic paraplegias are characterized by progessive spasticity and weakness of the legs. *Autosomal dominant spastic paraplegia-4* (SPG4) with relatively pure lower limb spastic paraplegia is caused by heterozygous mutations in the *SPAST* gene. Three new SPG4 patients were described who carried heterozygous deletions of various sizes, all including *SPAST* exon 17. All breakpoints occurred in *Alu* elements, in only one patient in *Alu* elements of the same family. All deletions displayed a microhomology at the breakpoint junction. In one patient, insertion of an *Alu* fragment was additionally found at the breakpoint junction. A microhomology-mediated deletion was proposed as the mechanism of exon loss. It was argued against non-allelic homologous recombination, because breakpoint *Alus* were of different families in 2 patients and sequence identity was <90% (Boone et al., 2011). Furthermore, 60 SPG4 patients were studied in leukocyte DNA for genetic rearrangements in *SPAST*. Some patients of this large study were published previously. Of the 54 CNVs analyzed, 70% appeared to have been mediated by an *Alu*-based mechanism. The *Alu*-mediated CNVs were in principle consistent with homologous recombination between non-allelic *Alus*. Based on short length (<300 bp) and low percent sequence identity (<91%) of *Alus*, the mechanism of *Alu*-*Alu* recombination was questioned. Alternative rearrangement mechanisms were discussed including microhomology-mediated DNA repair (Boone et al., 2014 and references therein). Moreover, 2 different CNVs in the *SPAST* gene of 2 separate German families were characterized. Both were deletions of different size and location and both carried insertions of different size at the breakpoint junction. The larger insertion matched an *Alu*Yb8 sequence while the smaller 15-bp insertion was in microhomology to a general *Alu* consensus sequence. Therefore, a mechanism with Alu retrotransposition (insertion) was proposed, followed by non-allelic homologous recombination (for the deletion with the larger insertion) or non-homologous end joining (for the deletion with the smaller insertion) (Jahic et al., 2016). Large deletions can also be found in the *SPG7* gene that is responsible for *spastic paraplegia type 7*, another form of autosomal recessive spastic paraplegia. Mother and son of a Spanish family with spastic paraplegia type 7 carried a complex rearrangement (the mother was homozygous and the son heterozygous for the rearrangement), which consisted of a deletion of *SPG7* exons 11–13, with the eventual 5′ breakpoint in an *Alu*Sq element, the 3′ breakpoint adjacent to a core chi-like sequence (gctgg), and insertion of a 8-bp fragment from an intermediate *Alu*Sx element at the breakpoint junction. The sequence gctgg is contained in the chi sequence (gctggtggg) and is also present in the 26-bp core region of some *Alu* elements. López et al. suggested that recombination between *Alu* elements and chi sequences can take place by formation of intrachromosomal loops and deletion of the intervening sequences. A microhomology-mediated deletion was proposed as additional rearrangement mechanism, because the breakpoint contained a 8-bp insertion (López et al., 2015). A similar picture with *Alu* microhomology-mediated exon deletion was observed in the gene *SPG11* in 3 patients with *autosomal recessive spastic paraplegia type 11* (Conceição Pereira et al., 2012; Table 1).

### Parkinson disease

Parkinson disease is diagnosed by presence of the clinical trias “rigor, tremor, and akinesia.” Dominance of one symptom of the Parkinsonian trias, co-morbidities, age of onset, and sporadic or familial occurence further classify this disease. Many candidate genes have been implicated in Parkinson disease and its variants (see OMIM). Mutations in the *PARK2* gene were made responsible for autosomal-recessive juvenile Parkinson disease. *PARK2* carries a high frequency of deletions. It was proposed that *PARK2* is located in a fragile site of the genome (Morais et al., 2016 and references therein). 17 Portugiese patients with autosomal-recessive juvenile Parkinson disease were reported with large homozygous or heterozygous *PARK2* deletions. PARK2 deletions were heterogeneous in size and location and sometimes contained an insertion fragment. 5′ and 3′ breakpoints occurred in different types of sequences: 2 *Alu* elements, 1 *Alu* and various unique sequences, 2 uniques sequences, 1 *Alu* and 1 DNA transposon, 1 LINE-1 and 1 LINE-2/LTR- transposon, etc. Several deletion mechanisms were discussed: nonallelic homologous recombination between *Alu* elements, nonhomologous end joining, and microhomology-mediated end joining. The variability in breakpoints and rearrangements was explained by the independence of recurrent events, which remains to be demonstrated. Only a few patients with homozygous deletions had identical breakpoints. A subset of these patients were cosanguinous and a founder mutation was made responsible for identical breakpoints (Morais et al., 2016). Mutations in the *DJ1* gene are implicated in autosomal recessive early-onset Parkinson disease (PARK7). One Dutch patient carried a homozygous genomic deletion of *DJ1* exons 1–5 with breakpoints occuring within 16 bp of sequence (microhomology) identical in 2 *Alu* elements flanking the deletion. An unequal crossing-over (or non-allelic homologous recombination) was the proposed genomic rearrangement mechanism (Bonifati et al., 2003; Table 1).

### Alzheimer disease

Mutations in the Amyloid Precursor protein (*APP*), Presenilin 1 (*PSEN1*) and Presenilin 2 (*PSEN2*) genes and duplications of the APP locus are the main causes of autosomal dominant early-onset *Alzheimer's disease*. One patient with a heterozygous *PSEN1* deletion of exons 9–10 was reported with breakpoints located in a 24 bp homologous sequence in 2 different *Alu* elements. A homologous *Alu*-mediated recombination was suggested, and causality was inferred from *in-vitro* experiments (Le Guennec et al., 2017; Table 1). Reduced A-to-I editing in Alzheimer's patients was found in prefrontal cortex at the GRIA2 Q/R site (99 vs. >99.9% in controls; Akbarian et al., 1995) and in hippocampus at various protein-recoding sites including GRIA3, GRIA4, GRI1, KCNA1, CACNA1D (Khermesh et al., 2016), and GRIA2 Q/R (Gaisler-Salomon et al., 2014; Table 1).

### Human epilepsy

Human epilepsy has as yet been associated with retrotransposon-mediated genetic rearrangements only in a few cases. Five patients have been reported with heterozygous *Alu*-mediated deletions in *ALDH7A1*, the gene responsible for pyridoxine-dependent epilepsy. This usually is a neonatal-onset epilepsy with various seizure types and unresponsiveness to standard anticonvulsants, responding only to immediate administration of pyridoxine hydrochloride. The dependence is permanent, and the interruption of daily pyridoxine supplementation leads to the recurrence of seizures. It is a metabolically caused epilepsy, because homozygous or compound heterozygous *ALDH7A1* mutations lead to enzyme deficiency and perturbation of the pipecolic acid pathway of lysine catabolism. In 2 sibelings with pyridoxine-dependent epilepsy, *ALDH7A1* exon 7 was heterozygously deleted and breakpoints were located in *Alus* of different families sharing 82% homology. A microhomology (overlapping identical sequence from the breakpoint *Alus*) of 11 bp was found at the breakpoint junction. A third patient carried a heterozygous *ALDH7A1* exon 18 deletion. Breakpoints occurred in 2 *Alu* elements that shared 85% homology. A 18-bp microhomology was found at the breakpoint junction. In 2 further patients, array CGH data indicated that breakpoints were located in intronic clusters of *Alu* elements. *Alu*-*Alu* recombination was the proposed rearrangement mechanism. Deletions were predicted to lead to a truncated protein in 3 patients and suspected to be detrimental, particularly as all patients additionally carried compound heterozygous *ALDH7A1* point mutations (Mefford et al., 2015). Early infantile epileptic encephalopathy-2 (EIEE2) with seizure onset in the first months of life and severe developmental delay has been associated with mutations in *CDKL5*. Three EIEE2 patients with variable deletions of *CDKL5* exons 1–4 were reported. In 2 patients the breakpoints were located in *Alu* elements of different families, and in one patient deletion between an *Alu*Jb and a unique sequence occurred. The breakpoint junctions of the *Alu*-*Alu* deletions contained perfect microhomology sequences (identical sequences 7–15-bp long). Microhomology-Mediated Break-Induced Replication/Fork Stalling and Template Switching was suggested as deletion mechanism (Erez et al., 2009). The human *CHNRA6* gene was shown to contain 2 *Alus* in tandem in its 5′ regulatory sequences that negatively controlled *CHRNA6* expression irrespectively of their dual or singulary presence and orientation (Ebihara et al., 2002). A-to-I editing alterations in humans with epilepsy have been reported (e.g., Krestel et al., 2013 and references therein), but more recent studies with an update on newly discovered protein-recoding sites, sites edited to low extent in combination with functional studies, or non-coding editing sites could not be identified in our data base search (but see Meier et al., 2016). While the function of RNA editing has to be analyzed in somatic tissue, i.e., brain, and in case of (mesial) temporal lobe epilepsy (TLE) in hippocampus, the studies regarding the contribution of retrotransposons to human epilepsy have as yet been performed in DNA from blood only. However, we are currently undertaking a study to analyze the contribution of retrotransposons and RNA editing to somatic mosaicism (see McConnell et al., 2013) in human hippocampi of TLE patients to identify additional genetic factors in a disease that could as yet be traced back to only few genetic variants in DNA from blood.

### Aicardi-goutières syndrome

*Aicardi-Goutières syndrome* is an early onset autoinflammatory disorder with spontaneous IFN production in the absence of virus infection (aseptic), particularly affecting the brain (cerebral atrophy, seizures, poor feeding, jitteriness, cerebral calcifications, white matter abnormalities) and skin. Mutations in *ADAR1* have been identified as one cause, although the mechanism(s) leading to the neurological manifestations remained unclear (Rice et al., 2012). ADAR1 deficiency has now been shown to cause spontaneous IFN-gamma production in human neuronal progenitor cells and apoptotic cell death (Chung et al., 2018). Given the fact that ADAR1 primarily edits *Alus* embedded in mRNAs, Aicardi-Goutières syndrome may be the prime example of an autoinflammatory disorder (with deficiently edited *Alus* resulting in an aseptic neuroinflammatory response), in which A-to-I editing of retrotransposons has gone awry.

## TDP-43 spectrum disorders, retrotransposons, A-to-I editing, and inflammation

Several recent results argue in favor of a cooperation of TDP-43 with ADARs. First, both are predominantly nuclear proteins, even if TDP-43 can also be encountered to a small percentage in the cytoplasmic fraction and here particularly bound to the 3′UTR of mRNAs. Second, both proteins bind to pre-mRNA. This is known for ADARs, and shown with predominant binding of TDP-43 to introns in the nuclear fraction of post-mortem human neocortex samples from 3 cognitively normal (healthy) and 3 patients with sporadic FTLD (Tollervey et al., 2011). Third, knockdown of TDP-43 in mouse lowers expression of the ADAR2 transcript (and many other transcripts; Polymenidou et al., 2011). Thus, a deficit in RNA editing secondary to altered TDP-43 levels is conceivable. Accordingly, hypoediting of GRIA2/Gria2 as the main substrate of ADAR2 in the findings of ALS patients and mouse models could be both the triggering event for TDP-43 cleavage with subsequent cytoplasmic aggregation (as mentioned in chapter 4; Yamashita et al., 2017) and the consequence of reduced ADAR2 expression due to nuclear TDP-43 depletion and cytoplasmic aggregation. The hypothesis of a secondary hypoediting of GRIA2/Gria2 seems to be supported by lowered ADAR2 levels in spinal cord motor neurons of ALS patients (Hideyama et al., 2012). A fourth piece of evidence for the relationship between ADAR-mediated A-to-I editing and altered TDP-43 levels originates from knockdown experiments in *Caenorhabditis elegans*, HeLa cells, and M17 neuroblastoma cells. siRNA knockdowns of the *Caenorhabditis elegans* ortholog TDP-1 and TDP-43 in HeLa and M17 cells not only showed an increase in transcripts from the human endogenous retrovirus of the K subfamily (HERVK), but also an increase of A-to-I edits in introns (in inverted repeats), and in 3′UTRs of mRNAs (Saldi et al., 2014). It can be interesting to investigate whether TDP-43 binds to, or close to, inverted intronic *Alu* elements and cooperates with ADARs to regulate translational efficiency, e.g., by modulating A-to-I editing-mediated backsplicing or alternative splicing including *Alu* exonization (Figure 4). The same may apply for TDP-43 binding to 3′UTRs and A-to-I editing of dsRNA structures in 3′UTRs. It is therefore tempting to speculate if reciprocal interaction between TDP-43 and ADARs also exists in the sense that primarily dysregulated A-to-I editing affects TDP-43 binding (Figure 4).

Another possibly interesting interaction exists between neurodegenerative diseases/TDP-43 spectrum disorders and neuroinflammation. Neuroinflammation was proposed to be one causative factor for the disease or at least for disease progression e.g., of Parkinson's or Alzheimer's disease (Whitton, 2007; Heneka et al., 2015). Current pathophysiological opinion is that e.g., Amyloid beta (in Alzheimer's) and alpha synuclein (in synucleinopathies including Parkinson's), together with sensing of ATP and DNA from damaged neurons via purinergic P2X7 receptors, activate microglia that are key cellular components of the neuroimmune system. Activated microglia in turn induce via NF-κB and other transcription factors the production of inflammatory cytokines and reactive oxygen species that progressively harm cholinergic or dopaminergic neurons, respectively (Glass et al., 2010). A new avenue in pathogenesis may arise from the potential direct activation of microglia by dysregulated TDP-43. Microglia have a “sensome” that refers to an unique grouping of transcripts coding for microglia receptors and transmembrane proteins used for sensing endogenous ligands and microbes. The sensome is more expressed in microglia compared to neurons and is supposed to signal proinflammatory microglial activation. The murine sensome with its top 100 transcripts is provided in Hickman et al. (2013). Of these 100 transcripts, 27 were significantly upregulated upon TDP-43 depletion following antisense nucleotide injection in striatum of normal adult mice. These upregulated transcripts were P2ry13, Tgfbr2, Tnfrsf1b, C3ar1, Ccrl2, C5ar1, Fcgr3, Fcer1g, Fcgr2b, Fcgr1, Ly86, Cd68, Trem2, Cd180, Tlr2, Tlr7, Cxcl16, Cd48, Cd74, Itgb5, Lgals9, Cd52, Icam1, Cd84, Ptprc, Cd22, Cd53). One transcript was significantly downregulated (Siglech) and the remaining 72% of transcripts had unchanged expression (Supplementary Information in Polymenidou et al., 2011). These results suggest microglial activation upon TDP-43 dysregulation (i.e., nuclear TDP-43 depletion). To test the hypothesis of transcriptional upregulation of the microglial sensome by nuclear TDP-43 depletion in patients, RNA-seq data are necessary that are not yet available from FTLD brains of patients, at least not in the Sequence Read Archive (SRA) of NCBI. The CLIP-seq data from Tollervey et al. cannot be used for this purpose as they had analyzed particular protein-RNA interactions (Tollervey et al., 2011). In the SRA, total RNA-seq data are however available of motor neuron populations isolated from sporadic ALS cases and controls (SRA accession SRP067645, project 306659) as well as from homogenized cervical spine sections of sporadic ALS and healthy control subjects (SRA accession SRP064478, project 297335). Thus, using these data it should be possible to evaluate the “upregulated TDP-43-microglia transcript” hypothesis, provided that the sensitivity of RNA-seq is high enough (i.e., enough reads have been generated). Otherwise, human microglia have to be isolated from post-mortem tissues (which is possible by FACS; e.g. Galatro et al., 2017), their RNA expression profiles determined and compared with murine data (Polymenidou et al., 2011).

## Immunogenicity of endogenous mRNAs with improperly edited inverted retrotransposons as additional cause for inflammation in neurological disease?

It is known for more than 50 years that the presence of foreign dsRNA in the cytoplasm of mammalian cells induces expression of interferon and thus activates an immune response (Lampson et al., 1967; Gantier and Williams, 2007). On the other hand, it is also known that cells produce large amounts of endogenous dsRNA. It was shown recently in human cells that mRNAs with embedded dsRNA structures are exported into the cytoplasm, that deficient A-to-I editing does not affect their cytosolic abundance, and that the purpose of A-to-I editing by ADAR1 rather seems to interfere with perfect matching of non-coding dsRNA and introduce formation of loop structures in order to avoid recognition by cytosolic soluble sensors of the interferon-mediated immune response such as MDA5; see above, chapter 3.2 (Liddicoat et al., 2015; George et al., 2016; Ahmad et al., 2018; Chung et al., 2018).

**Figure 5:**
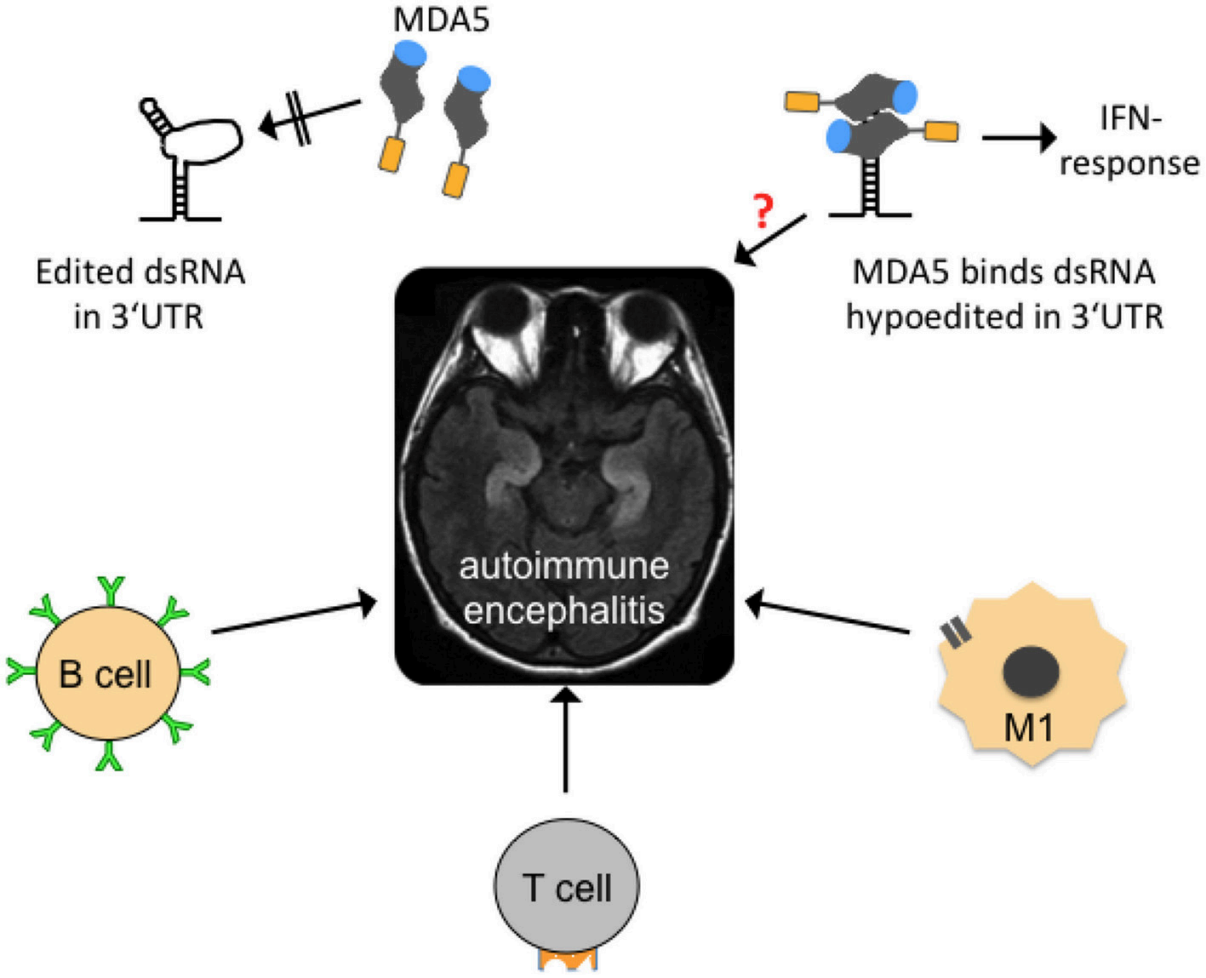
Possible mechanisms contributing to autoimmune encephalitides. In the center of the figure, an axial, T2-weighed magnetic resonance imaging section of a human brain is shown. The hyperintense (whitish “S”-shaped) structures represent inflammed, swollen hippocampi that are frequently affected in so-called autoimmune (limbic) encephalitis. Several forms are mainly B-lymphocyte- (B cell-) mediated with identification of a causative antibody (green). T-lymphocytes (T cell) and activated microglia (M1) may also contribute to neuroinflammation. Edited dsRNA in the 3′UTR of mRNAs may not be perfectly base-paired and thus contains loop structures that are not recognized by melanoma differentiation-associated gene 5 (MDA5), a cytoplasmic, soluble viral RNA receptor that activates a type I interferon mediated immune response (upper left graph). MDA5 consists of a N-terminal caspase recruitment domain (orange) a helicase domain (gray), and a C-terminal domain (blue; after Berke and Modis, 2012). Hypoedited and thus perfectly base-paired double-stranded RNA binds to the helicase and C-terminal domains and induces changes in the conformation and oligomerization of MDA5 that triggers an inflammatory response. An equivalent neuroinflammatory mechanism remains to be investigated in encephalitis patients (indicated by red question mark), but also in patients with neurodegenerative diseases, epilepsy, and other diseases where neuroinflammation is an important co-factor.

An association of hypoedited ds-*Alu* structures in 3′UTRs of mRNAs with neuroinflammation in TDP-43 spectrum disorders, neurodegenerative diseases in general (such as spinocerebellar ataxia, hereditary spastic paraplegia), and epilepsy has not been explored yet. The RNA editing status in neurological diseases has usually only been selectively examined with a focus on protein-recoding sites while data from sites in non-coding transcripts are largely missing (Table 1, Supplementary Text, Supplementary Table 1). Therefore, we can currently only speculate, which changes RNA editing undergoes at non-coding sites. Several neurological diseases (Huntington disease Akbarian et al., 1995; Alzheimer disease; Akbarian et al., 1995; Gaisler-Salomon et al., 2014; Khermesh et al., 2016; ALS; Kawahara et al., 2004) and epilepsy (e.g., Krestel et al., 2013), respond with both reduced and elevated A-to-I editing to their disease states and progression, with a tendency that reduced A-to-I editing levels at protein-recoding sites, compared to controls if they exist, prevail (for a recent review see Meier et al., 2016). If reduced editing of non-coding sites were also identified in inverted-repeat ds-*Alu* elements in 3′UTR of mRNAs this would represent an alternative hypothesis for neuroinflammation in these diseases. That is, because absence of ADAR1-mediated RNA modification would result in a structural loss of loops and gain of more perfectly matched dsRNA structures, which could be recognized by MDA5 that in turn would activate an immune response (Figure 5).

A relatively new field of autoimmune encephalitides now offers potential cure for some patients who may previously have been diagnosed with a psychiatric illness but also with epilepsy of unknown origin. Many of these acute-onset autoimmune encephalitides can now be diagnosed by detection of antibodies against glutamate receptors, GABA receptors, potassium channels or proteins of synaptic boutons such as LGI-1 and CASPR2, and others in blood or cerebrospinal fluid of patients (see e.g., Graus et al., 2016). Still, not all patients suspected to suffer from autoimmune encephalitis can be diagnosed with a particular causative antibody. These patients are thus probatorily treated with immune-modulatory drugs. If they respond to treatment (frequently only a few patients with partial response), their disease is usually termed steroid-responsive which covers all sorts of (mostly unknown) etiologies. Identification of a subset of mRNAs with deficiently edited 3′UTR dsRNA structures in the pool of >5% of mRNAs that are assumed to carry inverted-repeat ds-*Alu* structures in their 3′UTRs (Capshew et al., 2012) could reveal new pro-inflammatory causes, particularly in those steroid-responsive encephalopathies in which no causative autoimmune antibodies can as yet be identified.

## Conclusions and perspectives

### Transposable elements in the generation of genetic rearrangements

**Figure 6:**
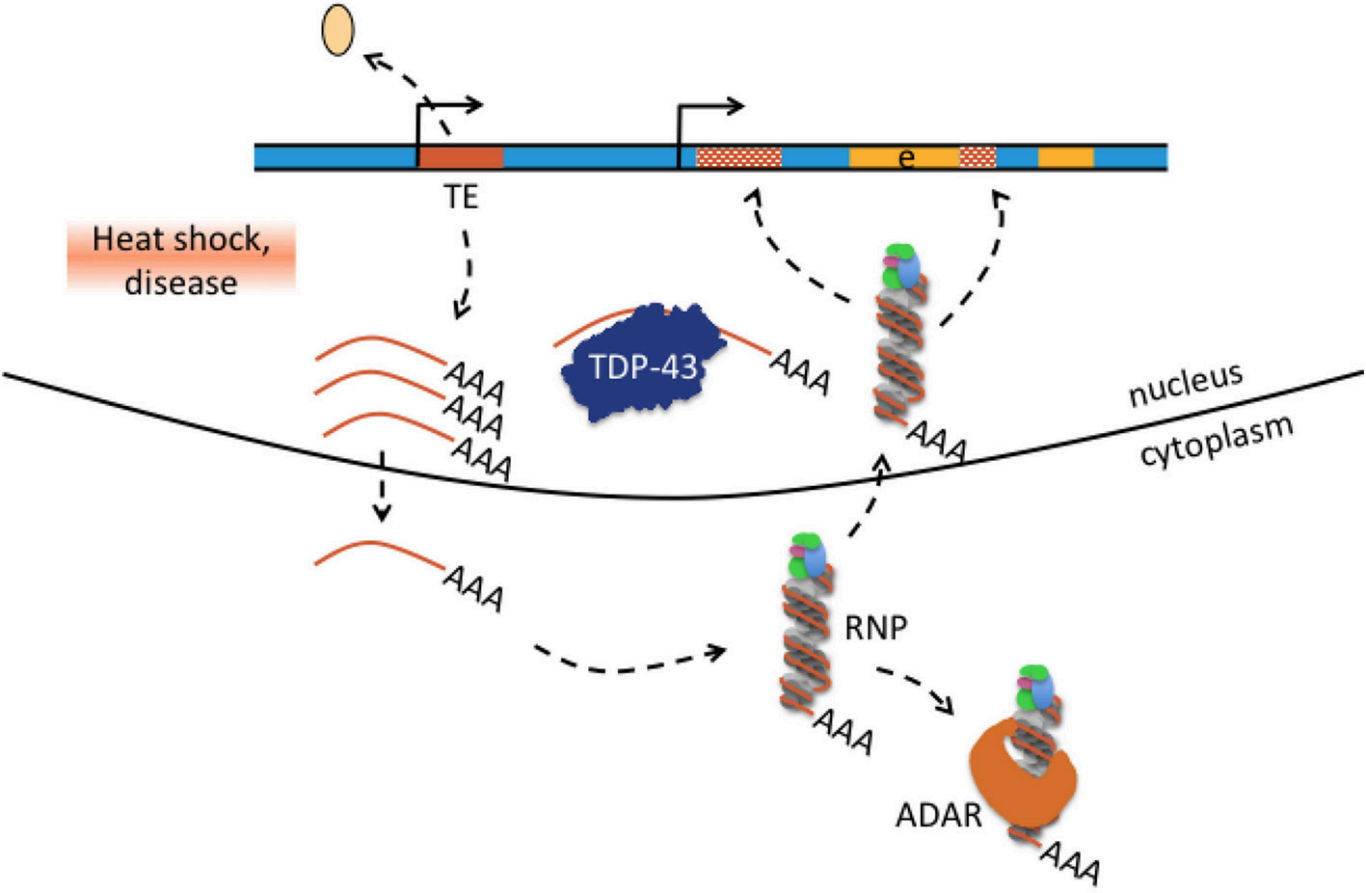
Hypothesis how transposable element-mediated genetic rearrangement could be counteracted by protein-RNA binding. Retrotransposon transcription from internal promoters can be activated by heat shock and disease as was for example reported in patients with amyotrophic lateral sclerosis-frontotemporal dementia (Prudencio et al., 2017; chapter 4). Activated TE transcription is indicated by detaching histone (beige oval), active promoter symbol, and multiple single-stranded mRNAs (brown lines with poly(A) tail). Single-stranded TE transcripts can be bound by TDP-43 or exported to the cytoplasm where they bind to ribonucleoprotein (RNP) particles depicted here as single-stranded RNA wrapped around RNP. Retrotransposon-RNP particles can be bound by ADAR or retransported into the nucleus where reverse-transcribed retrotransposons can integrate into the genome(hatched brown boxes). Excess TE transcripts could be scavanged by nuclear TDP-43 protein and cytosolic ADAR1 and thus may counteract TE mediated genetic rearrangement in neurological disease.

In most if not all neurological diseases cited in this review (Table 1, Supplementary Text, Supplementary Table 1), TEs were involved in genetic rearrangement and the association between rearrangement and disease cause was frequently convincing. RNA editing alterations in contrast have either not been investigated in many diseases or have almost exclusively been reported at protein-recoding sites. The few RNA editing events at protein recoding sites appeared seemingly detached from previous reports about the overwhelming majority of edits in non-coding *Alu* sequences, and from the genetic rearrangements in the diseases discussed here. In addition, A-to-I editing has as yet no function in the mechanisms that were proposed to cause these genetic rearrangements. These mechanisms briefly are non-allelic homologous recombination (NAHR), non-homologous end-joining (NHEJ), microhomology-mediated end-joining (MMEJ), and replication-based mechanisms that predominantly occur in the S phase of the cell cycle and which are fork stalling and template switching (FoSTeS) and microhomology-mediated break-induced replication (MMBIR) (Zhang et al., 2009; Ottaviani et al., 2014). The mechanism of *Alu* retrotransposition-mediated deletion adds to the repertoire of genetic rearrangements, particularly as *Alu* insertions do not have to occur at typical AT-rich L1 endonuclease cleavage sites but can also use single-strand genomic breaks and integrate without the target site duplications at its ends that are otherwise typical for canonical *Alu* insertions (Callinan et al., 2005). The genetic rearrangements of the diseases cited in this review carry sequence signatures of one or several of the above-mentioned mechanisms. The difficulties in defining the rearrangement mechanism were frequently mentioned in the publications describing them. Indeed, some rearrangements were proposed to have taken place by NAHR, but retrospectively carry sequence signatures that would also qualify them for NHEJ or *Alu* retrotransposition-mediated deletion (e.g., Kutsche et al., 2002; Bonifati et al., 2003; in the Supplementary Text). Many questions remain: the most important of them are how somatic rearrangements in the brain look like as all copy number variants in the neurological diseases described here have been exclusively described in DNA samples from EDTA-blood. Were so far only “tips of the icebergs” discovered by DNA analysis of blood samples - do neuronal genetic rearrangements look substantially different and contribute in as yet unexplored ways to disease pathogenesis - are genetic rearrangements ongoing in postmitotic neurons and which mechanisms apply? NAHR, NHEJ, MMEJ are the preferred methods of repair during the G1 and G2 phases of the cell cycle, while FoSTeS and MMBIR preferentially occur during the S phase. Which mechanisms do repair genomic deletions in differentiated neurons in the G0 phase, particularly those with microhomology or Alu fragment insertion at the breakpoint junctions and how are they initiated? In fact, increased expression of non-coding single-stranded transcripts and augmented genomic integration of *Alu* and L1 elements have been reported in several neurological diseases (e.g., in ALS-FTD, Prudencio et al., 2017; or Rett syndrome, Muotri et al., 2010, see Supplementary Text), and we propose that *Alu* (and possibly L1) retrotransposition-mediated deletion may be an accentuated mechanism for somatic genomic rearrangement. Accordingly, it may rather be the RNA-binding function of ADAR1 that offers some protection from *Alu* (and possibly L1) retrotransposition-mediated rearrangements than ADAR's enzymatic deamination function. There is evidence that *Alu* and LINE-1 retrotransposition is suppressed by ADAR1 through an RNA editing-independent mechanism that involves binding the L1/*Alu*-RNP complex (Orecchini et al., 2017b and references therein). Trapping of TE transcripts might also occur by other proteins. Actually, extensive binding of TDP-43 to non-coding single-stranded TE transcripts in brain tissue from FTD patients and in a disease mouse model was proposed to have a scavenging function (Li et al., 2012); see also Figure 6. If such a function turned out to be correct, it should be possible to detect TE transcripts in TDP-43 positive inclusions of classical TDP-43 spectrum disorders (which has not been reported until now).

### A-to-I editing of retrotransposons embedded in pol II generated mRNAs

A-to-I editing of intronic *Alu* elements can contribute to the generation of circular RNAs and thus to the regulation of translational efficiency as well as to the creation of alternative splice donor or acceptor sites and thus, via alternative splicing and exonisation, to new function (see chapter 3.2). Alu elements (without obvious editing) in the 5′UTR can regulate gene expression (see example with *CHNRA6* in chapter 4). The major function of ADAR1-mediated A-to-I editing of inverted-repeat *Alus* (and perhaps L1; Orecchini et al., 2017a and references therein) in the 3′UTR seems to be suppression (or regulation) of the endogenous IFN-mediated immune response (George et al., 2016; Mu et al., 2016; Ahmad et al., 2018; Chung et al., 2018). Thus, endogenous dsRNA structures emerge as a new factor in inflammation and in the context of this manuscript in neuroinflammation. The neuroimmune system is composed primarily of glial cells including astrocytes, microglia, oligodendrocytes, and mast cells originating from the hematopoietic lineage. Cellular components of the neuroimmune system contain cytosolic protein complexes, termed inflammasomes, that sense infectious or other host stimuli and initiate inflammatory responses through caspase activation (Mamik and Power, 2017). The activated inflammasomes e.g., in microglia are an emerging field with continuous identification of new triggers (e.g., Johann et al., 2015; Mamik and Power, 2017; TDP-43 as inflammasome trigger; Heneka et al., 2013; Zhao et al., 2015). Although hypoedited endogenous dsRNA structures are as yet not in the focus of attention as inflammatory trigger, more research in this line could answer several questions raised in this review, particularly as neuroinflammation is such an important co-factor in neurodegenerative diseases (Alzheimer's, Parkinson's, FTD, ALS etc.), epilepsy (e.g., Vezzani et al., 2011, 2013), and other diseases.

## Author contributions

HK wrote the manuscript. JM edited the manuscript. HK and JM produced the figures. All authors discussed the results and reviewed the manuscript.

### Conflict of interest statement

The authors declare that the research was conducted in the absence of any commercial or financial relationships that could be construed as a potential conflict of interest.

## Supplementary material

Supplementary Table 1 Inborn errors of metabolism and neurodevelopmental disorders. Diseases with neurological involvement as well as 2 types of spinocerebellar ataxia, spinal muscular atrophy, glioma, and schizophrenia are presented, for which human data about genetic rearrangements in association with transposable elements have been identified. For these diseases, human data about RNA editing were investigated and are presented. The very right column of the table contains major candidate genes (with chromosomal location given in brackets) that have been implicated in the respective diseases.
