# Supplementary material for: RNA Editing and Retrotransposons in Neurology
Source: Front Mol Neurosci. 2018 May 23;11:163. doi: 10.3389/fnmol.2018.00163 (PMC5974252; doi:10.3389/fnmol.2018.00163)
Supplement: Supplementary Table 1 — Inborn errors of metabolism and neurodevelopmental disorders. Diseases with neurological involvement as well as 2 types of spinocerebellar ataxia, spinal muscular atrophy, glioma, and schizophrenia are presented, for which human data about genetic rearrangements in association with transposable elements have been identified. For these diseases, human data about RNA editing were investigated and are presented. The very right column of the table contains major candidate genes (with chromosomal location given in brackets) that have been implicated in the respective diseases. [file Table_1.docx]

| **Disease** | **Transposable elements** (human data) | **RNA editing** (human data) | **Disease genes** |
| --- | --- | --- | --- |
| Acute intermittent porphyria | *Alu* insertion into exon 5 of *HMBS* gene with suppression of allelic expression in 6 family members (Mustajoki et al., 1999). | Not known | *HMBS* (11q) |
| Adrenoleukodystro-phy and Adrenomyelo-neuropathy | Large *ABCD1* deletion with breakpoints involving *Alu* elements (Kutsche et al., 2002) | Not known | *ABCD1* (Xq)*,*  www.x-ald.nl |
| Menkes disease | Exonic *Alu* insertion into *ATP7A* with alternative splicing (Gu et al., 2007) | Not known | *ATP7A* (Xq) |
| Wilson’s disease | Deletion and *Alu* exonization in *ATP7B* (Mameli et al., 2015) | Not known | *ATP7B* (13q) |
| CHARGE syndrome | *Alu*-mediated deletion deduced from poly(A) stretch at both breakpoints (Udaka et al. 2007) | Not known | *CHD7* (8q) |
| Hunter syndrome (mukopolysacchari-dosis II) | *Alu*-mediated deletion of *IDS* exon 8 combined with insertion of a partial *Alu*-L1 sequence (Ricci et al., 2003); Alu-mediated deletion of *IDS* exon 8 combined with insertion of a 49kb fragment that is normally found telomeric to IDS (Oshima et al., 2011) | Not known in brain, alternative U-to-C editing (enzyme unknown) in exon 1 of *IDS*, observed in fibroblasts from Hunter patients and controls (Lualdi et al., 2010, 2017)  A-to-I editing not known | *IDS* (Xq) |
| Walker Warburg syndrome | *Alu* insertion into exon 3 of *POMT1* with exon skipping (Bouchet et al., 2007) | Not known | *POMT1* (9q), *POMT2*, *POMGnT1*, *FKTN*, *FKRP* |
| Mucolipidosis II | *Alu*-mediated deletion of exon 19 of *GNPTAB* and alternative splicing of 3 different transcripts, 1 with *Alu* exonization (Coutinho et al., 2012); *Alu* retrotransposition into exon 5 with exon 5 skipping (Tappino et al., 2008) | Hypothesis of U-to-G RNA editing in exon 6 of *GNPTG* (mucolipidosis III gamma; Voltolini Velho et al., 2016)  A-to-I editing not known | *GNPTAB* (12q) |
| Maple syrup urine disease | 1 patient with homozygous deletion in *BCKDHA* between an *Alu* in intron 1 and intron 4 without repetitive elements (Quental et al., 2008); 2 patients with homozygous deletions in *DBT* between L1 in intron 10 and *Alu* in 3’UTR (Chi et al., 2003); 10 patients with identical *DBT* deletion as described by Chi et al. (Silao et al., 2004); 1 patient with heterozygous deletion in *DBT* between *Alu* in intron 6 and non-homologous region in exon 11 (Herring et al., 1992) | Not known | *BCKDHA* (19q), *BCKDHB*, *DBT* (1p) |
| Congenital disorder of glycosylation | *Alu*-mediated deletion in *PMM2*, combined with insertion of an *Alu* fragment (Schollen et al., 2007) | Not known | *PMM2* (16p),  http://www.euroglycanet.org/  home.html |
| Tetrahydrobiopterin deficiency | 1 patient with heterozygous deletion in *PTS* intron 2, leading to *Alu* exonization, 1 patient with homozygous point mutation in *PTS* intron 1, resulting in LINE-2 exonization (Meili et al., 2009) | Not known | *PTS* (11q) |
| Tay Sachs disease | *Alu*-mediated deletion of *HEAXA* promoter region and exon 1 (Myerowitz et al., 1986, 1987) | Not known | *HEXA* (15q) |
| Lesch-Nyhan disease | *Alu*-mediated deletions of various parts of *HPRT1* (Mizunuma et al., 2000; Mizunuma et al., 2001; Brooks et al., 2001; Tvrdik et al., 1998; Marcus et al., 1993) | Not known | *HPRT1* (Xq) |
| Rett syndrome | *Alu*-mediated deletion in *MECP2* between chi sequences and *Alu* elements (Laccone et al., 2004); increased L1 retrotransposition in hippocampi, indicating propensity for genomic rearrangement (Muotri et al., 2009) | Not known | *MECP2* (Xq) |
| Septo-optic dysplasia | 1 patient with homozygous *Alu* insertion into exon 3 of *HESX1* (Sobrier et al., 2005); 1 patient with homozygous *Alu*-mediated recombination and deletion of entire *SOX2* (Suzuki et al., 2014) | Not known | *HESX1* (3p), *SOX2* (3q) |
| Neurofibromatosis  type 1 | Case report: *Alu* insertion in *NF1* intron 5 with exon 6 skipping and protein truncation (Wallace et al., 1991);  altered splicing of *NF1* by *Alu* or L1 insertions in 18 patients (Wimmer et al., 2011); most non-recurrent CNVs in *NF1* in a cohort of 87 NF1 patients due to DNA replication-based mechanisms and minority due to *Alu*-based rearrangements (Hsiao et al., 2015) | C-to-U RNA editing of NF1 by APOBEC (Cappione et al., 1997)  A-to-I editing not known | *NF1* (17q) |
| Autosomal recessive spinocerebellar ataxia-1 | 1 patient with *SETX* L1 insertion and exon 12 skipping, 1 patient with *Alu*-mediated deletion of *SETX* exons 12-14, 2 patients with L1 and *Alu*-mediated deletion of *SETX* exons 11-15 (Bernard et al., 2009); 1 patient with *Alu*-mediated in tandem duplication of *SETX* exons 7-10 (Arning et al., 2008) | Not known | *SETX* (9q) |
| Spinocerebellar ataxia type 10 | ATTCT repeat expansion within nested repetitive sequences including *Alu* and L1 elements located in *ATXN10* intron 9 (Kurosaki et al., 2009) | Not known | *ATXN10* (22q) |
| Huntington’s disease  (HD) | *Alu* in intron near 5’ of *ADD1* gene; unrelated to HD (Goldberg et al., 1993; Hutchinson et al, 1993) | Striatum: GRIA2 Q/R site editing 95% (Akbarian et al., 1995) | *HTT* (4p) |
| Spinal muscular atrophy type I | *Alu*-mediated deletion of exons 5+6 of *SMN1* (Wirth et al., 1999) | Not known | *SMN1* (5q) |
| Spinal muscular atrophy with respira-tory distress type 1 | *Alu*-mediated deletion of exons 3-7 of *IGHMBP2* (Guenther et al., 2004) | Not known | *IGHMBP2* (11q) |
| Schizophrenia | *Alu* element within recombination hotspot of *GABRB2* serves as recombining sequence for rearrangement (Ng et al., 2010); genomic L1 increased in copy numbers and inserted in regions enriched with genes associated with schizophrenia and bipolar disorder (Bundo et al., 2014) | Controversial deficiency of AMPA receptor GRIA2 editing in prefrontal cortex (Akbarian et al., 1995; Silberberg et al., 2012);  controversial HTR2C editing (Sodhi et al., 2001; Dracheva et al., 2003); no alterations at any protein-coding A-to-I editing sites (Zhu et al., 2012; Lyddon et al., 2012) | *GABRB2* (5q),  for further candidate genes see OMIM |
| Glioma | Glioma risk factor *EGFR* variant III mutation consisting of deletion of exons 2-7 can be *Alu*-mediated (Frederick et al., 2000) | Deficient A-to-I editing of: GRIA2, GRIK2, HTR2C (Maas et al., 2001; Cenci et al., 2008); CDC14B (Galeano et al., 2013), ADAR2 (Wei et al., 2014), miR-376a (Choudhury et al., 2012), miR-222/221 and miR-21 (Tomaselli et al., 2015) | *IDH, ATRX, TERT, EGFR* (7p)*, BRAF* |
