# Supplementary material for: RNA Editing and Retrotransposons in Neurology
Source: Front Mol Neurosci. 2018 May 23;11:163. doi: 10.3389/fnmol.2018.00163 (PMC5974252; doi:10.3389/fnmol.2018.00163)
Supplement: Supplementary file 2 [file Data_Sheet_1.docx]

**Supplementary Text**

**Diseases with central nervous system involvement that have been associated with transposable elements**

Acute intermittent porphyria is a genetic metabolic disorder with early onset and variable symptoms including urinary and gastrointestinal symptoms, muscle weakness, and central neurologic symptoms such as seizures, anxiety, headache, hallucinations, confusion, and depression. It is caused by partial deficiency of the enzyme porphobilinogen deaminase, encoded by the *HBMS* (*PBGD*) gene. Three of 4 members of a Finnish family with acute intermittent porphyria carried an *Alu* insertion in exon 5 of *HBMS*, as well as 3 other family members who were clinically asymptomatic. All patients carrying the *Alu* insertion had low PBGD enzymatic activity in their erythrocytes, one of the diagnostic hallmarks of acute intermittent porphyria (Mustajoki et al., 1999; Supplementary Table). *Alu* insertion suppressed expression of the allelic transcript and was thus was found to be responsible for the low PGPD enzymatic activity. *In vitro* expresison of the HBMS cDNA carrying the *Alu* insertion in exon 5 resulted in a truncated protein with lack of any enzymatic activity. RNA editing alterations have as yet not been reported in acute intermittent porphyria.

Adrenoleukodystrophy (ALD) is a peroxisomal inborn error of metabolism with various ages of onset, neurologic symptoms, and caused by sequence alterations in the *ABCD1* gene. Adrenomyeloneuropathy is the milder form of ALD with later onset, progressive neuropathy and paraparesis, and cerebral involvement in approx. 45% of cases. The majority of *ABCD1* mutations are single nucleotide variants. Large deletions have been described, notably in 2 ALD patients with homozygous partial deletion of the *ABCD1* gene and breakpoints within *Alu* elements. One patient had in addition to the deletion an *Alu* sequence inserted at the breakpoint junction. A link between *Alu*-mediated genomic rearrangement, deficient ALD protein, and thus ALD was proposed (Kutsche et al., 2002). RNA editing has as yet not been reported to modify ALD (Supplementary Table).

Menkes and Wilson disease are both genetic diseases with abnormal copper metabolism, onset in infancy, and sequence variants in copper-transporting ATPases (*ATP7A* on Xq21.1 in Menkes disease and *ATP7B* on 13q14.3 in Wilson’s disease). Reduced enzymatic activity results in a variety of symptoms. Menkes disease is the more severe of the 2 diseases with shorter life expectancy. While symptoms in Wilson’s disease arise from excess copper deposition in the body including the brain, the neurologic symptoms in Menkes disease probably also arise from the failure to transfer copper into copper-dependent enzymes (for further clinical and genetic description see OMIM). A case report of an infant diagnosed with Menkes disease identified an *Alu* insertion into exon 9 of the *ATP7A* gene in whole blood genetic analysis that resulted in skipping of the whole exon 9 in mRNA and translation of a truncated protein. The zygosity of the insertion was not mentioned nor could it be deduced from the presented data. The work identified new exonic splicing enhancers in the *Alu* insertion, but did not explain the mechanism of alternative ATP7A mRNA splicing in this patient. It was stated that exonic splicing enhancers recruit components of the core splicing machinery to nearby splice sites (Gu et al., 2007; Supplementary Table). Thus, the association between Menkes disease and deficient enzymatic ATP7A activity due to *Alu*-mediated alternative splicing is plausible, while the impact of the new exonic splicing enhancer sequence motifs on alternative splicing in this patient remains to be proven. A description of a child with Wilson’s disease identified a large homozygous deletion in the *ATP7B* gene with activation of 3 cryptic splice sites, resulting in exonization of a fragmented cryptic *Alu* element into the mature mRNA (Mameli et al., 2015). The alternatively spliced mRNA contained a stop codon in the *Alu* exon and likely produced a truncated non-functional protein. Both parents carried the same deletion in heterozygosity, but it was not mentioned whether *Alu* exonization also occurred in them. The mechanisms of deletion and *Alu* exonization were explained as follows: A Tigger DNA transposon was identified at the proximal breakpoint, suggesting that the deletion occurred at a recombination hot spot. *Alu* exonization resulted from deletion of a canonical acceptor splice site in intron 1 together with slowed transcription, favoring splicing at cryptic splice sites. A truncated protein was predicted. Nevertheless, the patient was a responder to zinc therapy and remained clinically asymptomatic during the observation period of the study (Mameli et al., 2015). RNA editing in humans has not yet been associated with Menkes and Wilson disease (Supplementary Table).

CHARGE syndrome [acronym for coloboma, heart defects, atresia (of nasal choanae), retardation, genital and ear abnormalities] is characterized by a pattern of congenital anomalies and is caused by heterozygous mutations in the gene encoding the chromodomain helicase DNA-binding protein 7(*CHD7*). An *Alu*-mediated heterozygous deletion of exons 8-12 was deduced from poly(A) stretches found at breakpoints in introns 7 and 12 of *CHD7*. It was not commented how the deletion affected the patient’s phenotype (Udaka et al., 2007; Supplementary Table).

X-linked recessive Hunter syndrome (mucopolysaccharidosis II) is a lysosomal storage disease with infantile onset and is caused by the deficient or absent enzyme iduronate-2-sulfatase leading to accumulation of heparan sufate as well as dermatan sulfate in all organs of the body. The responsible *IDS* gene is rich in multiple interspersed repeats (Ricci et al., 2003). One case report identified a heterozygous deletion between a 5’ breakpoint in intron 7 and the 3’ breakpoint within an *Alu*Sc element in intron 8 of the *IDS* gene, combined with the 157-bp insertion of a sequence that partially corresponds to an *Alu* element adjacent to an L1 that can normally be found centromeric to *IDS* on the X chromosome (Ricci et al., 2003). The male patient inherited this genomic rearrangement from his clinically asymptomatic mother. A homologous unequal recombination between the *Alu* element in *IDS* intron 8 and an *Alu* element centromeric to the *IDS* gene was proposed. In a second male patient with Hunter syndrome who also inherited the genetic rearrangement from his clinically asymptomatic mother, 5’ and 3’ breakpoints occurred in a low-copy repeat in *IDS* intron 7 and in an *Alu*Sc element, flanked by 2 L1 in intron 8, respectively, and led to exon 8 deletion. At the breakpoint junction, an insertion of a ~49 kb fragment was found that is normally located telomeric to the *IDS* gene (Oshima et al., 2011; Supplementary Table). A complex rearrangement was proposed with homologous recombination between 2 low-copy repeats (one in *IDS* intron 7, a second in the *IDSP1* pseudogene located ~25kb telomeric to *IDS*) and 2 L1-*Alu*-L1 elements (one in intron 8, the second ~75kb telomeric to *IDS*). Why the particular *Alu* element in intron 8 was chosen as 3’ breakpoint remained unclear because intron 8 contained 17 *Alu* elements and many of them were flanked by LINE elements (Oshima et al., 2011). A-to-I editing has not been reported in Hunter syndrome. One case report of 2 male patients with Hunter syndrome suggested that U-to-C RNA editing corrected the coding variant c.22C>T in exon 1 of the *IDS* gene into the normal mRNA sequence; several other explanations such as genomic mosaicism of the cell culture were ruled out. Their hypothesis was based on the presence of both mutant and wildtype mRNAs in cDNA from fibroblast or lymphoblast cultures of the respective patients, while genomic DNA only carried the *IDS* c.22C>T variant (Lualdi et al., 2010; Supplementary Table). The same group subsequently transfected mutant IDS cDNA into normal fibroblasts and showed that alternative U-to-C editing occurred in a certain percentage of transcripts. The responsible RNA editing enzyme was not specified (Lualdi et al., 2017).

Walker Warburg syndrome is an early lethal disease associated with brain (lissencephaly, hydrocephalus, cerebellar malformations) and eye abnormalities, and muscular dystrophy, mainly due to enzymatic deficiency of O-mannosyltransferase, encoded by *POMT1*. In a report of 3 unrelated fetuses, aborted for Walker Warburg syndrome, an *Alu* insertion into *POMT1* exon 3 was found, in one fetus as homozygous insertion and in the other 2 as heterozygous insertions. In tissue (not specified if also neural tissue) from each of the 3 fetuses, the insertion resulted in skipping of exon 3 and a frameshift with premature stop. The *Alu* element was inserted at a typical L1 endouclease recognition site (Bouchet et al., 2007).

Mucolipidosis II is a metabolic disorder with early onset, reduced life span, and involvement of neurologic symptoms such as psychomotor retardation. It belongs to the group of lysosymal storage diseases. Here, the defective enzyme N-acetylglucosamine-1- phosphotransferase, encoded by *GNPTAB*, fails to target specific proteins to lysosomes with the consequence that molecules cannot be normally broken down anymore. A case with genomic rearrangement, breakpoints in *Alu* elements in introns 18 and 19, and deletion of *GNPTAB* exon 19 was described. The deletion was homozygous and created 3 abnormal transcripts, one with skipping of exon 19, one with skipping of exons 19 and 20, and one with skipping of exon 19 and exonization of an *Alu* element in intron 18. The mechanisms of alternate skipping of exon 19 or exons 19 and 20 were not discussed. Thus, it remained unclear how the molecular machinery decided which of these 3 types of mRNA to transcribe and to which extent. The creation of a pseudo-exon in the 3rd type of transcript was explained by the presence of a sequence element at the 5’ splice site of the *Alu* element needed for the generation of an alternatively spliced *Alu* exon (Coutinho et al., 2012; Sorek et al., 2004). Another patient with Mucolipidosis II showed an *Alu* insertion in *GNPTAB* exon 5, which resulted in a transcript lacking exon 5. The *Alu* insertion generated new exonic splicing enhancers that were made responsible for the alternative splicing (Tappino et al., 2008). Putative U-to-G RNA editing in exon 6 of the *GNPTG* transcript was suggested as mechanism explaining the sequence differences between genomic DNA and cDNA from cultured fibroblasts of 2 sibelings with mucolipidosis III gamma, a rare autosomal-recessive disease caused by pathogenic sequence variants in the *GNPTG* gene (Voltolini et al., 2016). The homozygous sequence variant in exon 6 of *GNPTG* from genomic DNA was predicted to lead to a premature stop, but low levels of non-mutated GNPTG mRNA were found. The enzyme performing this putative U-to-G conversion was not characterized. A putative conversion of U to G at the mRNA level in kainate GRIK3 (GluR7) transcripts from a human fetal brain library was mentioned only once in the literature (Nutt et al., 1994). Whether this type of RNA editing really exists remains to be shown. A-to-I RNA editing has not yet been reported in Mucolipidosis II or III gamma (Supplementary Table).

Maple syrup urine disease is an autosomal recessive metabolic disorder, affecting branched-chain amino acids. It is one type of organic acidemia, often accompanied with severe brain damage, particularly in the classic neonatal severe form. Maple syrup urine disease is caused by impaired function of the branched-chain alpha-keto acid dehydrogenase complex, a mitochondrial multienzyme complex formed by three catalytic components: a decarboxylase composed of alpha (*BCKDHA* gene) and beta (*BCKDHB* gene) subunits; a dihydrolipoyl transacylase (*DBT* gene), and a dihydrolipoamide dehydrogenase (*DLD* gene). In one case report, a homozygous deletion within the *BCKDHA* gene was made responsible for the disease. The 5’ breakpoint was located in an *Alu* element within intron 1, while the 3’ breakpoint in intron 4 did not contain any repetitive element. A non-homologous recombination was suggested to underlie the deletion of exons 2-4 (Quental et al., 2008). Two children of the Austronesian aboriginal tribe Paiwan in Taiwan with maple syrup urine disease were described to carry homozygous deletions of exon 11 of the *DBT* gene, with the deletion occuring between the 5’ breakpoint in a L1 element in intron 10 and the 3’ breakpoint within an *Alu* element in the 3’ UTR of the gene. No significant homology between the L1-PA7 and *Alu*Sx elements was observed; still a non-homologous recombination event was proposed (Chi et al., 2003). A causality between the deletion and the disease was proposed as the truncated protein lost its catalytic domain. Notably, 10 patients of Filipinian origin had an identical deletion in the *DBT* gene as the 2 patients published by Chi et al., with the 5’ breakpoint in an L1 element in intron 10 and the 3’ breakpoint in an *Alu* element in the 3’ UTR following exon 11 (Silao et al., 2004). Intron 11 with the remaining L1 sequence was in fact fused with the remaining *Alu* sequence in the 3’ UTR in the following way: GAAGGAGGCAGTCTGT**GT**GGTGGTGGGT (GT in bold indicates overlapping 2 nucleotides (microhomology) from intron 11 and the 3’ UTR. Three sibelings from one Filipino family and 4 individuals from 4 further families had homozygous deletions, while 3 individuals from yet another 3 families had heterozygous deletions (Silao et al., 2004). Finally, a patient presented with yet another type of deletion in the *DBT* gene. The heterozygous deletion spanned from the 5’ breakpoint within an *Alu* element in intron 6 to a 3’ breakpoint within exon 11. In compound heterozygosity, a coding single nucleotide variant was found that destroyed the splice donor site of intron 8 and resulted in skipping of either exon 8 or exons 8-10. All transcripts produced truncated proteins, but still the patient was responsive to thiamin treatment, implicating that the truncated proteins were able to exert some function (Herring et al., 1992).

Congenital disorder of glycosylation is another inborn error of metabolism. The synthesis of glycoconjugates by phospho-mannomutase 2 (encoded by *PMM2*) is defective, resulting in a multisystem disorder with severe encephalopathy, peripheral neuropathy, retinitis pigmentosa, peculiar subcutaneous fat distribution, hypogonadism, and typically reduced life span. An infant with severe congenital disorder of glycosylation was found to be compound heterozygous for a mutation p.V44A (c.131T>C) and exon 8 deletion with concomitant insertion of a 260bp-long *Alu* element at the breakpoint junction in the *PMM2* gene. A causal relationship between the genetic alterations and the phenotype was proposed because enzymatic phospho-mannomutase activity was reduced to residual 10% (Schollen et al., 2007).

Tetrahydrobiopterin (BH4) deficiency is a rare metabolic disorder with infantile onset and is caused by autosomal recessive mutations in the gene encoding the enzyme 6-pyruvoyl-tetrahydropterin synthase (*PTS*) that is involved in the synthesis or regeneration of BH4. BH4 is a cofactor for phenylalanine hydroxylase (resulting in hyperphenylalaninemia in case of BH4 deficiency), tyrosine hydroxylase, and tryptophan hydroxylase, the latter 2 of which are involved in neurotransmitter synthesis. BH4 deficiency can result in delayed development including intellectual disability, movement disorders, behavioral problems, and seizures. In one case of BH4 deficiency, the poly(A) tail of an *Alu* element and a 3’ splice site normally used during splicing were heterozygously deleted in *PTS* intron 2. This brought a new favorable branch point sequence, located upstream to the deletion, in closer proximity to a 3’ splice site within the 5’ end of the *Alu* element, resulting in the exonization of the *Alu* element. Two transcripts were identified; one was correctly spliced and one contained the new pseudo-exon. A causal relationship between the genomic rearrangement and the phenotype was postulated because PTS enzymatic activity was reduced 2-30-fold, depending on the tissue that was examined. A second patient in the same study was even more severely affected by 10-30-fold reduction of PTS activity in red blood cells/fibroblasts. Her phenotype was traced back to a homozygous single nucleotide variant in intron 1, located downstream to an LINE-2 A element, leading to the use of cryptic splice sites with exonization of a LINE-2 fragment (Meili et al., 2009; Supplementary Table). Another patient with BH4 deficiency and a similar genetic rearrangement with *Alu* exonization as published previously (Meili et al., 2009) was described more recently (Brasil et al., 2011).

Further inborn errors of metabolism with neurological involvement have been associated with retrotransposons, but not yet with A-to-I editing (Supplementary Table): *Alu*-mediated genomic rearrangement in Tay-Sachs disease (inborn disorder of gangliosidosis metabolism with neurologic involvement; Myerowitz and Hogikyan, 1987) and Lesch-Nyhan disease (inborn disorder of purin metabolism with neurologic involvement; Mizunuma et al., 2000; Mizunuma et al., 2001; Brooks et al., 2001; Tvrdik et al., 1998; Marcus et al., 1993).

Rett syndrome is a developmental disorder with initially normal development and secondary regression, autism features and symptoms including unusual body proportions, muscular hypotonia, dystonia or increased tonus resulting in impaired walking ability, ataxia, extrapyramidal symptoms, and bruxism. Rett syndrome is caused by mutations in X-chromosomal *MECP2*. Mutations cannot be identified in about 20-30% of cases, but another possiblity is the occurence of larger deletions that may not be detected by routine methods (Laccone et al., 2004). *MECP2* is highly enriched for repeat elements inlcuding *Alu* and L1. Seven patients with Rett syndrome were described who had *MECP2* deletions with one breakpoint located in an *Alu* element. Eight out of the same series of 11 patients had one breakpoint in a deletion-prone region of *MECP2*. This deletion-prone region contains a chi sequence (gctggtggg), and gctgg is also present in the 26-bp core region of some *Alu* elements. Laccone et al. suggested that recombination between *Alu* elements and chi sequences can take place by formation of intrachromosomal loops and deletion of the intervening sequences. The authors further suggested that *MECP2* deletions can now be added to the rising number of *Alu*-mediated deletions resulting in monogenic diseases (Supplementary Table). As L1 promoter activity is controlled by MeCP2 and DNA methylation, together with other transcription factors, L1 retrotransposon activity was investigated with Taqman-based quantitative real-time PCR in hippocampi and hearts of 5 deceased Rett syndrome patients and 5 controls. Hippocampi of Rett syndrome patients had increased L1 copy numbers, indicating an increased susceptibility for genomic rearrangement also by L1 retrotransposition (Muotri et al., 2010). RNA editing has as yet not been identified as additional regulatory mechanism in the pathogenesis of Rett syndrome.

Septo-optic dysplasia is a rare congenital malformation syndrome defined by any combination of optic nerve hypoplasia, pituitary gland hypoplasia, and midline abnormalities of the brain, including absence of the corpus callosum and septum pellucidum (Dattani et al., 1998). The diagnosis is made when 2 or more features of the classic triad are present. Approximately 30% of patients have full manifestations. Sequence variants in *HESX1*, *OTX2*, *SOX2*, and *PAX6* were implicated in this syndrome. One adolescent with coloboma and panhypopituitarism carried a homozygous *Alu* insertion into exon 3 of the *HESX1* gene. This insertion generated 2 types of alternatively spliced mRNAs, one with skipping of exon 3 and another with skipping of exon 2 and 3. The authors argued for a causal relationship between the genomic insertion and the phenotype as the functional homeobox domain of HESX1 was lost (Sobrier et al., 2005). One child with developmental retardation, microphthalmia, seizures, and anterior pituitary deficiency had a homozygous 2.3 Mb large deletion on chromosome 3q that contained the single exon *SOX2* gene (Suzuki et al., 2014). An *Alu*-mediated recombination was assumed because the breakpoint junction showed *AluY* sequences on both sides.

Neurofibromatosis type 1 (NF1) is a neurocutaneous developmental disorder, typically with infantile onset, with multiple tumors along the nervous system, skin abnormalities, mental disabilities and epilepsy. NF1 is caused by genetic alterations in *NF1*, which typically are microdeletions. *Alu* and L1 insertions into the *NF1* gene have also been reported (Wallace et al., 1991; Wimmer et al., 2011). It was assumed that 0.4% of *NF1* mutations are caused by retrotransposon insertions (Wimmer et al., 2011). This estimation was based on analysis of DNA and RNA from patients’ blood leukocytes. The true positive rate might be higher if brain tumors were genetically analyzed. The association between between retrotransposon insertions and neurofibromatosis type 1 is quite strong: All the so far reported *Alu* and L1 insertions have led to altered splicing with partial or entire exon skipping, resulting in truncated proteins or in proteins lacking a certain number of amino acids (Wallace et al., 1991; Wimmer et al.; 2011). The choice of alternative or cryptic splice sites remained unexplained in all but 4 cases in which retrotransposon insertion destroyed the 3’ splice site (Wimmer et al., 2011). Intragenic *NF1* rearrangements in a cohort of 87 unrelated patients were analyzed in DNA samples from EDTA blood. In 33 of 87 individuals (38%), the origin for the rearrangement involved *Alu* elements: *Alu*-mediated non-allelic homologous recombination was proposed in 18 patients (in 2 patients most likely followed by an *Alu*Y insertion) and in one case an intronic *Alu* insertion, followed by *Alu*-*Alu*-mediated recombination (blunt end breakpoint junction). The predominant mechanisms for intragenic *NF1* rearrangement were however proposed to be fork stalling and template switching and/or microhomology-medated break-induced recombination (both DNA-replication-based mechanisms) (Hsiao et al., 2015). Alternative C-to-U RNA editing by APOBEC-1 was observed in exon 23-1 (or 23a, alternatively spliced exon 23 that is divided by an intron; Li et al., 1995) of NF1 transcripts with a trend towards higher editing levels in more malignant tumors (1.5-2.0 % in nontumor tissues and non-NF1 individuals versus 4-17% in neural tumors; Supplementary Table). C-to-U edited NF1 mRNA leads to a truncated form of the NF1 protein neurofibromin, but the consequences in normal tissue and tumors remain to be determined. Exons 21-27a of *NF1* are thought to interact with Ras protein, thereby inactivating Ras-mediated signal transduction and exerting tumor-suppressor acivity (Cappione et al., 1997; Supplementary Table). A PubMed search for "neurofibromatosis type 1“ or "NF1“ and "A-to-I editing“ or "ADAR“ remained negative.

The rare variant autosomal recessive spinocerebellar ataxia-1 (SCAR1) is caused by mutations in the gene senataxin (*SETX*) that codes for a helicase. Four juvenile to jung adult patients were reported, one with heterozygous L1 insertion into *SETX* exon 12 with skipping of exon 12, one with heterozygous deletion of exons 12-14 between 2 breakpoints located in *Alu* elements, and 2 patients with deletion of exons 11-15 between breakpoints in a L1 and an *Alu* element (Bernard et al., 2009). Another patient was genetically diagnosed with SCAR1 at adult age. A heterozygous duplication of exons 7-10 in tandem was found flanked by *Alu* element sequences on both sides, leading to a truncated protein due to introduction of a premature stop codon. In addition, a compound heterozygous mutation in exon 8 was found that also generates a premature stop codon. Both genetic alterations were predicted to explain the patient’s phenotype (Arning et al., 2008).

Spinocerebellar ataxia type 10 is caused by an unstable ATTCT pentanucleotide repeat expansion in intron 9 of the *ATXN10* gene, but the mechanism leading to disease has not fully been clarified because the mutant allele is transcribed to similar extent as the wildtype allele and regularly spliced. Notably, the ATTCT repeat expansion is located within nested repetitive sequences consisting of L1, *Alu*, and ERVK (endogenous retrovirus group K) sequences (Kurosaki et al., 2009 and references therein). It was suggested that the ATTCT repeats originated from a poly(A) tail of an *Alu* element and its junction that together with nucleotide substitutions had inserted into *ATXN10* intron 9 (Kurosaki et al., 2009 and references therein). No interaction of this genetic rearrangement or spinocerebellar ataxia type 10 with RNA editing has been reported yet (Supplementary Table).

Huntington disease is a choreatic movement disorder with histopathological focus in the basal ganglia and particularly in caudate nucleus. Huntington disease is caused by increased CAG repeats in the *huntingtin* (*HTT*) gene. In 2 Huntington’s disease families, the insertion of an *Alu* element into the *ADD1* gene within the possible genomic Huntington disease locus was reported (Goldberg et al., 1993), before *HTT* was identified. Following publication of the *HTT* gene sequence (The Huntington’s disease collaborative Research group, 1993), the hypothesis of *Alu* insertion into the genomic Huntington disease locus as a cause of disease could not be validated for Huntington disease (Hutchinson et al., 1993). Thus, Huntington disease is as yet not related to transposable elements. However, deficient A-to-I editing at the Q/R site of AMPA receptor GRIA2 subunits was found in striatum of Huntington disease patients (95% editing versus 99.5% in controls; Akbarian et al., 1995; Supplementary Table) with a possible role of neuronal hyperexcitability and neurodegeneration in the course of disease.

Autosomal recessive spinal muscular atrophy (SMA) type I is a motor neuron disease with infantile onset, in which only the secondary motor neuron in the spinal cord degenerates, resulting in flaccid para- or tetraparesis. It is caused by homozygous loss of the *SMN1* gene. 80 – 98% of SMA patients show homozygous deletion of *SMN1* exon 7, resulting in a truncated less stable SMN protein. Absence of SMN1 is partially compensated for by its homolog SMN2, but *SMN2* itself frequently shows deletion of exon 7. Phenotype severity is determined by mutations in *SMN1* and *SMN2* and can be influenced by *SMN2* copy number. Two independent patients carried a similar ~6 kb deletion of exons 5 and 6 of the *SMN1* gene. One of these patients was sequenced in more detail: Breakpoints occurred in 2 antisense-oriented *Alu* elements in introns 4 and 6, respectively, that shared > 80% homology. The breakpoint junction revealed a microhomology of 27 bp and was located 4 nucleotides upstream to a 26 bp *Alu* core sequence in intron 6 that was considered responsible for homologous and nonhomologous recombinations (Wirth et al. 1999). The SMA region on chromosome 5q13 contains 5 times more *Alu* elements than the genome in general (Wirth et al., 1999 and references therein). The same *Alu* element that is located in intron 6 of *SMN1* and *SMN2* can not only serve as a breakpoint but can also exonize. Exon 6B-containing SMN1 and SMN2 transcripts were found in a mouse model with mild SMA and also in all normal human tissues examined with highest expression in brain (Seo et al., 2016). Protein expression of exon 6B-containing transcrips was lower in fibroblasts of type I SMA patients than in an unrelated SH-SY5Y cell line with neuron-like characteristics (Seo et al., 2016). The suggested purpose of SMN6B protein was to suppress skipping of exon 7 in *SMN2* and thus a compensatory mechanism for *SMN1* genetic alterations (Seo et al., 2016). A similar picture of genomic rearrangement was found in a case with infantile autosomal recessive spinal muscular atrophy with respiratory distress type 1 (SMARD1) characterized by progessive distal muscle weakness and difficulty to breathe. SMARD1 is caused by homozygous or compound-heterozygous *IGHMBP2* mutations, with IGHMBP2 being a helicase. In the published case, the 5’ and 3’ breakpoints occurred in *Alu* elements in intron 2 and 7, respectively, with heterozygous deletion of *IGHBP2* exons 3-7. Non-allelic homologous recombination was suggested, as the 2 intronic *Alu* elements were homologous to 81%. The authors could not comment on the genotype-phenotype relationship of this patient with an unsusually mild course of disease because the patient additionally had a previously unreported missense mutation in the second *IGHMBP2* allele (Guenther et al., 2004; Supplementary Table).

Schizophrenia is a disorder of thought and sense of self. For a clinical description and the many candidate genes and regions inolved in this disease, we refer to the OMIM website. Recently, a recombination hotspot in the *GABRB2* gene has been associated with schizophrenia. The recombination hotspot spans from *GABRB2* intron 8 to 9 and contains 29 single nucleotide polymorphisms (SNP) as well as an *Alu* element in intron 8. Determination of recombination rates showed recombinations in the sequences flanking the *Alu* element rather than in the *Alu* itself, which is consistent with a role of the *Alu* element to serve as recombining sequence for homologous recombination. The co-occurence of schizophrenia risk-conferring haplotypes and protective haplotypes observed in controls argues for a high rate of recombination-selection for a phenotype at this hotspot. Intronic *Alu* elements may function again in recombination; in this case for SNP haplotypes (Ng et al., 2010). Genomic LINE-1 elements were significantly increased in copy number in prefrontal cortex of schizophrenia patients, intraindividually in comparison to the L1 number in liver, and interindividually in comparison to prefrontal cortex of controls (Bundo et al., 2014). To identify the genomic locations of the insertions, 3 schizophrenia patients and 3 controls were chosen for whole genome sequencing. Using a gene ontology approach, the genes affected by insertions were neuronal function-related, such as "synapse“ and "protein phosphorylation“. In addition, genomic regions with insertions were enriched in genes associated with schizophrenia and bipolar disorder, although no detailed gene list was provided (Bundo et al., 2014). These data only provide a weak causal genotype-phenotype relationship. Deficient A-to-I editing was found in the prefrontal cortex of patients with schizophrenia (99% editing versus >99.9% in controls; Akbarian et al., 1995). In contrast, subsequent work did not identify deficient GRIA2 Q/R editing in prefrontal cortex of patients with schizophrenia (Silberberg et al., 2012). Investigation of A-to-I editing at sites in the serotonin receptor HTR2C in prefrontal cortex was also controversial: One study with 15 patients with schizophrenia and treated with neuroleptic medication did not reveal any significant differences in comparison to matched controls (Dracheva et al., 2003), while a previous study with 5 patients and controls found differentially edited HTR2C isoforms in schizophrenia patients and controls (Sodhi et al., 2001; Supplementary Table). In both studies, Brodman area 46 was prepared, but different results may have arisen from the number of clones analyzed: While >30 clones per patient were analyzed in Dracheva et al. 2003, only 10 clones per patient were analyzed in the Sodhi study. More recent studies (Lyddon et al., 2012; Zhu et al., 2012) then rather ruled out significant alterations at the GRIA2, GRIA3, GRIA4, GRIK1, GRIK2, HTR2C, KCNA1, and other protein-coding A-to-I editing sites in prefrontal cortex of schizophrenia patients.

Glioblastoma multiforme is the most aggressive variant of gliomas, which are primary brain tumors arising from the glial cell lineage, and is associated with short-term survival. Glioblastoma multiforme was not found to contain novel somatic TE (L1, *Alu*, ERV) insertions in 16 different genomes (Lee et al., 2012). This is in contrast to the observation that retrotransposition is frequent in other types of cancer including squamous lung, head and neck, colorectal, and endometrial carcinomas (Helman et al., 2014; Lee et al., 2012). This discrepancy may be explained by the fact that glioblastoma arises in >90% from glial cells, and retrotransposition in the brain may occur primarily in the neuronal lineage (Richardson et al., 2014). On the other hand, it was shown that somatic L1 insertions occurred at a rate of ~0.58-1 events per cell in both glia and neurons from human hippocampus and frontal cortex and affect at least 36% of the cells in the healthy adult brain (Erwin et al., 2016). TE insertions may thus be of less importance in glioblastoma than e.g. larger deletions involving transposable elements. In this regard may the following studies be of note: Epidermal growth factor receptor (EGFR) has an important function in gliomas because EGFR variant III with deletion of exons 2-7 is frequently found in glioblastoma and *EGFR* gene amplification (overexpression) may increase the risk factors for poor prognosis of glioblastoma patients (Brandner et al., 2018; Frederick et al., 2000). *EGFR* contains many intronic *Alu* repeats, and in 6 glioblastoma patients out of a series of 20, *Alu* elements were involved in the deletions found in *EGFRvIII*. It was concluded that *Alu* repeats are involved in *EGFR* rearrangements and may cause the expression of *EGFRvIII*. As truncated EGFRvIII protein is constitutively active (Kuan et al., 2001), it and indirectly *Alu*-mediated rearrangement may contribute to glioma growth. Deficient RNA editing at various sites has been described in glioma (Maas et al., 2001; Cenci et al., 2008; Galeano et al., 2013; Wei et al., 2014). Not only protein-recoding sites were affected but also editing sites in non-coding transcripts such as miR-376a (Choudhury et al., 2012) as well as miR-222/221 and miR-21 (Tomaselli et al., 2015).

**References**

Akbarian, S., Smith, M. A., and Jones, E. G. (1995). Editing for an AMPA receptor subunit RNA in prefrontal cortex and striatum in Alzheimer's disease, Huntington's disease and schizophrenia. *Brain Res.* 699, 297-304.

Arning, L., Schöls, L., Cin, J., Souquet, M., Epplen, J. T., and Timmann, D. (2008). Identification and characterisation of a large Senataxin (SETX) gene duplication in ataxia with ocular apraxia type 2 (AOA2). *Neurogenetics* 9, 295–299.

Bernard, V., Minnerop, M., Bürk, K., Kreuz, F., Gillessen-Kaesbach, G., and Zühlke C. (2009). Exon deletions and intragenic insertions are not rare in ataxia with oculomotor apraxia 2. *BMC Med. Genet.* 10, 87.

Bouchet, C., Vuillaumier-Barrot, S., Gonzales, M., Boukari, S., Le Bizec, C., Fallet, C., et al. (2007). Detection of an Alu insertion in the POMT1 gene from three French Walker Warburg syndrome families. *Mol. Genet. Metab.* 90, 93–96.

Brandner, S., and Jaunmuktane, Z. (2018). Neurological update: gliomas and other primary brain tumours in adults. *J. Neurol.* 265, 717–727.

Brasil, S., Viecelli, H. M., Meili, D., Rassi, A., Desviat, L. R., Pérez, B., et al. (2011). Pseudoexon exclusion by antisense therapy in 6-pyruvoyl-tetrahydropterin synthase deficiency. *Hum. Mutat.* 32, 1019-1027.

Brooks, E. M., Branda, R. F., Nicklas, J. A., and O'Neill, J. P. (2001). Molecular description of three macro-deletions and an Alu-Alu recombination-mediated duplication in the HPRT gene in four patients with Lesch-Nyhan disease. *Mutat. Res.* 476, 43-54.

Bundo, M., Toyoshima, M., Okada, Y., Akamatsu, W., Ueda, J., Nemoto-Miyauchi, T., et al. (2014). Increased L1 retrotransposition in the neuronal genome in schizophrenia. *Neuron* 81, 306–313.

Cappione, A. J., French, B. L., and Skuse, G. R. (1997). A Potential Role for NFl mRNA Editing in the Pathogenesis of NFl Tumors. *Am. J. Hum. Genet.* 60, 305-312

Cenci, C., Barzotti, R., Galeano, F., Corbelli, S., Rota, R., Massimi, L., et al. (2008). Down-regulation of RNA editing in pediatric astrocytomas: ADAR2 editing activity inhibits cell migration and proliferation. *J. Biol. Chem.* 283,7251-7260.

Chi, C. S., Tsai, C. R., Chen, L. H., Lee, H. F., Mak, B. S., Yang, S. H., et al. (2003). Maple syrup urine disease in the Austronesian aboriginal tribe Paiwan of Taiwan: a novel DBT (E2) gene 4.7 kb founder deletion caused by a non-homologous recombination between LINE-1 and Alu and the carrier-frequency determination. *Eur. J. Hum. Genet.* 11, 931-936.

Choudhury, Y., Tay, F. C., Lam, D. H., Sandanaraj, E., Tang, C., Ang, B. T., et al. (2012). Attenuated adenosine-to-inosine editing of microRNA-376a* promotes invasiveness of glioblastoma cells. *J. Clin. Invest.* 122, 4059-4076.

Coutinho, M. F., da Silva Santos, L., Lacerda, L., Quental, S., Wibrand, F., Lund, A. M., et al. (2012). Alu–Alu recombination underlying the first large genomic deletion in GlcNAc-phosphotransferase alpha/beta (GNPTAB) gene in a MLII alpha/beta patient. *JIMD Rep.* 4, 117-124.

Dattani, M. T., Martinez-Barbera, J.-P., Thomas, P. Q., Brickman, J. M., Gupta, R., Martensson, I.-L., et al. (1998). Mutations in the homeobox gene HESX1/Hesx1 associated with septo-optic dysplasia in human and mouse. *Nat. Genet.* 19, 125-133.

Dracheva, S., Elhakem, S. L., Marcus, S. M., Siever, L. J., McGurk, S. R., and Haroutunian, V. (2003). RNA editing and alternative splicing of human serotonin 2C receptor in schizophrenia. *J. Neurochem.* 87, 1402-1412.

Frederick, L., Eley, G., Wang, X. Y., and James, C. D. (2000). Analysis of genomic rearrangements associated with EGFRvIII expression suggests involvement of Alu repeat elements. *Neuro Oncol.* 2, 159-163.

Galeano, F., Rossetti, C., Tomaselli, S., Cifaldi, L., Lezzerini, M., Pezzullo, M., et al. (2013). ADAR2-editing activity inhibits glioblastoma growth through the modulation of the CDC14B/Skp2/p21/p27 axis. *Oncogene* 32, 998-1009.

Goldberg, Y. P., Rommens, J. M., Andrew, S. E., Hutchinson, G. B., Lin, B., Theilmann, J., et al. (1993). Identification of an Alu retrotransposition event in close proximity to a strong candidate gene for Huntington's disease. *Nature* 362, 370-373.

Gu, Y. H., Kodama, H., Watanabe, S., Kikuchi, N., Ishituka, I., Ozawa, H. et al. (2007). The first reported case of Menkes disease caused by an Alu insertion mutation. *Brain Dev.* 29, 105-108.

Guenther, U. P., Schuelke, M., Bertini, E., D’Amico, A., Goemans, N., Grohmann, K., et al. (2004). Genomic rearrangements at the IGHMBP2 gene locus in two patients with SMARD1. *Hum. Genet.* 115, 319-326.

Helman, E., Lawrence, M. S., Stewart, C., Sougnez, C., Getz, G., and Meyerson, M. (2014). Somatic retrotransposition in human cancer revealed by whole-genome and exome sequencing. *Genome Res.* 24, 1053-1063.

Herring, W. J., McKean, M., Dracopoli, N., and Danner, D. J. (1992). Branched chain acyltransferase absence due to an Alu-based genomic deletion allele and an exon skipping allele in a compound heterozygote proband expressing maple syrup urine disease. *Biochim. Biophys. Acta* 1138, 236-242.

Hsiao, M. C., Piotrowski, A., Callens, T., Fu, C., Wimmer, K., Claes, K. B., et al. (2015). Decoding NF1 Intragenic Copy-Number Variations. *Am. J. Hum. Genet.* 97, 238-249.

Hutchinson, G. B., Andrew, S. E., McDonald, H., Goldberg, Y. P., Graham, R., Rommens, J. M., et al. (1993). An AIu element retroposition in two families with Huntington disease defines a new active AIu subfamily. *Nucleic Acids Res.* 21, 3379-3383.

Kuan, C. T., Wikstrand, C. J., and Bigner, D. D. (2001). EGF mutant receptor vIII as a molecular target in cancer therapy. *Endocrine-Related Cancer* 8, 83–96.

Kurosaki, T., Matsuura, T., Ohno, K., and Ueda, S. (2009). Alu-mediated acquisition of unstable ATTCT pentanucleotide repeats in the human ATXN10 gene. *Mol. Biol. Evol.* 26, 2573–2579.

Kutsche, K., Ressler, B., Katzera, H-G., Orth, U., Gillessen-Kaesbach, G., Morlot, S., Schwinger, E., et al. (2002). Characterization of breakpoint sequences of five rearrangements in L1CAM and ABCD1 (ALD) Genes. *Hum. Mutat.* 19, 526-535.

Laccone, F., Jünemann, I., Whatley, S., Morgan, R., Butler, R., Huppke, P., et al. (2004). Large deletions of the MECP2 gene detected by gene dosage analysis in patients with Rett syndrome. *Hum. Mutat.* 23, 234-244.

Li, Y., O'Connell, P., Breidenbach, H. H., Cawthon, R., Stevens, J., Xu, G., et al. (1995). Genomic organization of the neurofibromatosis 1 gene (NF1). *Genomics* 25; 9-18.

Lee, E., Iskow, R., Yang, L., Gokcumen, O., Haseley, P., Luquette 3rd, L. J., et al; Cancer Genome Atlas Research Network. (2012). Landscape of Somatic Retrotransposition in Human Cancers. *Science* 337, 967-971.

Lualdi, S., Tappino, B., Di Duca, M., Dardis, A., Anderson, C. J., Biassoni, R., et al. (2010). Enigmatic In Vivo Iduronate-2-Sulfatase (IDS) Mutant Transcript Correction to Wild-Type in Hunter Syndrome. *Hum. Mutat.* 31, E1261-E1285.

Lualdi, S., Del Zotto, G., Zegarra-Moran, O., Pdemento, N., Corsolini, F., Bruschi, M., et al. (2017). In vitro recapitulation of the site-specific editing (to wild-type) of mutant IDS mRNA transcripts, and the characterization of IDS protein translated from the editedmRNAs. *Hum. Mutat.* 38, 849-862.

Lyddon, R., Navarrett, S., and Dracheva, S. (2012). Ionotropic glutamate receptor mRNA editing in the prefrontal cortex: no alterations in schizophrenia or bipolar disorder. *J. Psychiatry Neurosci.* 37, 267-272.

Maas, S., Patt, S., Schrey, M., and Rich, A. (2001). Underediting of glutamate receptor GluR-B mRNA in malignant gliomas. *Proc. Natl. Acad. Sci. U S A* 98, 14687-1492.

Mameli, E., Lepori, M. B., Chiappe, F., Ranucci, G., Di Dato, F., Iorio, R., et al. (2015). Wilson's disease caused by alternative splicing and Alu exonization due to a homozygous 3039-bp deletion spanning from intron 1 to exon 2 of the ATP7B gene. *Gene* 56, 276-279.

Marcus, S., Hellgren, D., Lambert, B., Fällström, S. P., and Wahlström, J. (1993). Duplication in the hypoxanthine phosphoribosyl-transferase gene caused by Alu-Alu recombination in a patient with Lesch Nyhan syndrome. *Hum. Genet.* 90, 477-482.

Meili, D., Kralovicova, J., Zagalak, J., Bonafé, L., Fiori, L., Blau, N., et al. (2009). Disease-causing mutations improving the branch site and polypyrimidine tract: pseudoexon activation of LINE-2 and antisense Alu lacking the Poly(T)-tail. *Hum. Mutat.* 30, 823-831.

Mizunuma, M., Fujimori, S., Kaneko, K., and Kamatani, N. (2000). Deletion in the hypoxanthine phosphoribosyltransferase gene caused by Alu-Alu recombination in two Japanese patients with Lesch-Nyhan syndrome. *Adv. Exp. Med.* Biol. 486, 23-27.

Mizunuma, M., Fujimori, S., Ogino, H., Ueno, T., Inoue, H., and Kamatani, N. (2001). A recurrent large Alu-mediated deletion in the hypoxanthine phosphoribosyltransferase (HPRT1) gene associated with Lesch-Nyhan syndrome. *Hum. Mutat.* 18, 435-443.

Muotri, A. R., Marchetto, M. C., Coufal, N. G., Oefner, R., Yeo, G., Nakashima, K., et al. (2010). L1 retrotransposition in neurons is modulated by MeCP2. *Nature* 468, 443-446.

Mustajoki, S., Ahola, H., Mustajoki, P., and Kauppinen, R. (1999). Insertion of Alu element responsible for acute intermittent porphyria. *Hum. Mutat.* 13, 431-438.

Myerowitz, R., and Hogikyan, N. D. (1987). A deletion involving Alu sequences in the beta-hexosaminidase alpha-chain gene of French Canadians with Tay-Sachs disease. *J. Biol. Chem.* 262, 15396-15399.

Ng, S. K., Lo, W. S., Pun, F. W., Zhao, C., Yu, Z., Chen, J., et al. (2010). A recombination hotspot in a schizophrenia-associated region of GABRB2. *PLoS One* 5, e9547.

Nutt, S. L., Hoo, K. H., Rampersad, V., Deverill, R. M., Elliott, C. E., Fletcher, E. J., et al. (1994). Molecular characterization of the human EAA5 (GluR7) receptor: a high-affinity kainate receptor with novel potential RNA editing sites. *Receptors Channels* 2, 315-326.

Oshima, J., Lee, J. A., Breman, A. M., Fernandes, P. H., Babovic-Vuksanovic, D., Ward, P. A., et al. (2011). LCR-initiated rearrangements at the IDS locus, completed with Alu-mediated recombination or non-homologous end joining. *J. Hum. Genet.* 56, 516-523.

Quental, S., Martins, E., Vilarinho, L., Amorim, A., and Joao Prata, M. (2008). Maple syrup urine disease due to a new large deletion at BCKDHA caused by non-homologous recombination. *J. Inherit. Metab. Dis.* 31, S457–S460.

Ricci, V., Regis, S., Di Duca, M., and Filocamo, M. (2003). An Alu-mediated rearrangement as cause of exon skipping in Hunter disease. *Hum. Genet.* 112, 419-425.

Richardson, S. R., Morell, S., and Faulkner, G. J. (2014). L1 retrotransposons and somatic mosaicism in the brain. *Annu. Rev. Genet.* 48, 1-27.

Schollen, E., Keldermans, L., Foulquier, F., Briones, P., Chabas, A., Sánchez-Valverde, F., et al. (2007). Characterization of two unusual truncating PMM2 mutations in two CDG-Ia patients. *Mol. Genet. Metab.* 90, 408-413.

Seo, J., Singh, N. N., Ottesen, E. W., Lee, B. M., and Singh, R. N. (2016). A novel human-specific splice isoform alters the critical C-terminus of Survival Motor Neuron protein. *Sci. Rep.* 6, 30778.

Silao, L.T., Padilla, C. D., and Matsuo, M. (2004). A novel deletion creating a new terminal exon of the dihydrolipoyl transacylase gene is a founder mutation of Filipino maple syrup urine disease. *Mol. Genet. Metabol.* 81, 100-104.

Silberberg, G., Lundin, D., Navon, R, and Öhman, M. (2012). Deregulation of the A-to-I RNA editing mechanism in psychiatric disorders. *Hum. Mol. Genet.* 21, 311-321

Sobrier, M. L., Netchine, I., Heinrichs, C., Thibaud, N., Vié-Luton, M. P., Van Vliet, G., et al. (2005). Alu-element insertion in the homeodomain of HESX1 and aplasia of the anterior pituitary. *Hum. Mutat.* 25, 503.

Sodhi, M. S., Burnet, P. W., Makoff, A. J., Kerwin, R. W., and Harrison, P. J. (2001). RNA editing of the 5-HT(2C) receptor is reduced in schizophrenia. *Mol. Psychiatry* 6, 373-379.

Sorek, R., Lev-Maor, G., Reznik, M., Dagan, T., Belinky, F., Graur, D., et al. (2004). Minimal Conditions for Exonization of Intronic Sequences: 5’ Splice Site Formation in Alu Exons. *Mol. Cell* 14, 221–231.

Suzuki, J., Azuma, N., Dateki, S., Soneda, S., Muroya, K., Yamamoto, Y., et al. (2014). Mutation spectrum and phenotypic variation in nine patients with SOX2 abnormalities. *J. Hum. Genet.* 59, 353-356.

Tappino, B., Regis, S., Corsolini, F., and Filocamo, M. (2008). An Alu insertion in compound heterozygosity with a microduplication in GNPTAB gene underlies Mucolipidosis II. *Mol. Genet. Metabol.* 93, 129-133.

The Huntington’s disease collaborative Research group. (1993). *Cell* 72, 971-983.

Tomaselli, S., Galeano, F., Alon, S., Raho, S., Galardi, S., Polito, V. A., et al. (2015). Modulation of microRNA editing, expression and processing by ADAR2 deaminase in glioblastoma. *Genome Biol.* 16, 5.

Tvrdik, T., Marcus, S., Hou, S. M., Fält, S., Noori, P., Podlutskaja, N., et al. (1998). Molecular characterization of two deletion events involving Alu-sequences, one novel base substitution and two tentative hotspot mutations in the hypoxanthine phosphoribosyltransferase (HPRT) gene in five patients with Lesch-Nyhan syndrome. *Hum. Genet.* 103, 311-318.

Udaka, T., Okamoto, N., Aramaki, M., Torii, C., Kosaki, R., Hosokai, N., et al. (2007). An Alu Retrotransposition-Mediated Deletion of CHD7 in a Patient With CHARGE Syndrome. *Am. J. Med. Genet.* 143A, 721-726.

Voltolini Velho, R., Ludwig, N. F., Alegra, T., Sperb-Ludwig, F., Ruas Guarany, N., Matte, U., et al. (2016). Enigmatic in vivo GlcNAc-1-phosphotransferase (GNPTG) transcript correction to wild type in two mucolipidosis III gamma siblings homozygous for nonsense mutations. *J. Hum. Genet.* 61, 550-560.

Wallace, M. R., Andersen, L. B., Saulino, A. M., Gregory, P. E., Glover, T. W., and Collins, F. S. (1991). A de novo Alu insertion results in neurofibromatosis type 1. *Nature* 353, 864-866.

Wimmer, K., Callens, T., Wernstedt, A., and Messiaen, L. (2011). The NF1 Gene Contains Hotspots for L1 Endonuclease-Dependent De Novo Insertion. *PLoS Genet.* 7, e1002371.

Wirth, B., Herz, M., Wetter, A., Moskau, S., Hahnen, E., Rudnik-Schöneborn, S, et al. (1999). Quantitative Analysis of Survival Motor Neuron Copies: Identification of Subtle SMN1 Mutations in Patients with Spinal Muscular Atrophy, Genotype-Phenotype Correlation, and Implications for Genetic Counseling. *Am. J. Hum. Genet.* 64, 1340-1356.

Wei, J., Li, Z., Du, C., Qi, B., Zhao, X., Wang, L., et al. (2014). Abnormal expression of an ADAR2 alternative splicing variant in gliomas downregulates adenosine-to-inosine RNA editing. *Acta Neurochir. (Wien)* 156, 1135-1142.

Zhu, H., Urban, D. J., Blashka, J., McPheeters, M. T., Kroeze, W. K., Mieczkowski, P., et al. (2012). Quantitative analysis of focused A-to-I RNA editing sites by ultra-high-throughput sequencing in psychiatric Disorders. *PLoS One* 7, e43227.
